# Neuroinvasive West Nile Infection Elicits Elevated and Atypically Polarized T Cell Responses That Promote a Pathogenic Outcome

**DOI:** 10.1371/journal.ppat.1005375

**Published:** 2016-01-21

**Authors:** Eddie A. James, Theresa J. Gates, Rebecca E. LaFond, Shinobu Yamamoto, Chester Ni, Duy Mai, Vivian H. Gersuk, Kimberly O’Brien, Quynh-Anh Nguyen, Brad Zeitner, Marion C. Lanteri, Philip J. Norris, Damien Chaussabel, Uma Malhotra, William W. Kwok

**Affiliations:** 1 Benaroya Research Institute at Virginia Mason, Seattle, Washington, United States of America; 2 Blood Systems Research Institute, San Francisco, California, United States of America; 3 Departments of Laboratory Medicine and Medicine, University of California, San Francisco, San Francisco, California, United States of America; 4 Virginia Mason Medical Center, Seattle, Washington, United States of America; 5 Department of Medicine, University of Washington, Seattle, Washington, United States of America; NIH, UNITED STATES

## Abstract

Most West Nile virus (WNV) infections are asymptomatic, but some lead to neuroinvasive disease with symptoms ranging from disorientation to paralysis and death. Evidence from animal models suggests that neuroinvasive infections may arise as a consequence of impaired immune protection. However, other data suggest that neurologic symptoms may arise as a consequence of immune mediated damage. We demonstrate that elevated immune responses are present in neuroinvasive disease by directly characterizing WNV-specific T cells in subjects with laboratory documented infections using human histocompatibility leukocyte antigen (HLA) class II tetramers. Subjects with neuroinvasive infections had higher overall numbers of WNV-specific T cells than those with asymptomatic infections. Independent of this, we also observed age related increases in WNV-specific T cell responses. Further analysis revealed that WNV-specific T cell responses included a population of atypically polarized CXCR3+CCR4+CCR6- T cells, whose presence was highly correlated with neuroinvasive disease. Moreover, a higher proportion of WNV-specific T cells in these subjects co-produced interferon-γ and interleukin 4 than those from asymptomatic subjects. More globally, subjects with neuroinvasive infections had reduced numbers of CD4+FoxP3+ Tregs that were CTLA4 positive and exhibited a distinct upregulated transcript profile that was absent in subjects with asymptomatic infections. Thus, subjects with neuroinvasive WNV infections exhibited elevated, dysregulated, and atypically polarized responses, suggesting that immune mediated damage may indeed contribute to pathogenic outcomes.

## Author Summary

Most West Nile virus (WNV) infections are asymptomatic, but some lead to neuroinvasive disease with symptoms ranging from disorientation to paralysis and death. Epidemiologic evidence suggests that neuroinvasive disease is a consequence of poor viral control, as the risk of neurological symptom is highest in immune compromised individuals. However, conflicting evidence suggests that neurologic symptoms may arise because of immune mediated damage. We demonstrate that subjects with neuroinvasive West Nile Virus infections have exaggerated and atypical responses to the virus. Subjects with neuroinvasive infections had higher numbers of WNV-responsive cells and these cells had more potent and diverse functional responses. In particular, we observed that subjects with neuroinvasive infections had a significantly increased population of atypically polarized T cells, whose presence was highly correlated with a globally upregulated transcript profile. Thus, we conclude that immune mediated damage may indeed contribute to neurologic symptoms and pathogenic outcomes in the setting of WNV infection.

## Introduction

Since its emergence in 1999, West Nile virus (WNV) has become a leading cause of encephalitis in North America. Seasonal outbreaks in various states have led to thousands of documented cases of WNV infection and perhaps millions of undocumented cases, based on serological estimates [1]. Epidemiological data indicate that the majority of WNV infections are essentially asymptomatic [2]. However, a minor yet significant percentage of infections lead to neuroinvasive disease [3]. The symptoms elicited by neuroinvasive WNV infection can include ocular manifestations, muscle weakness, cognitive impairment, tremors, flaccid paralysis, and death, making this virus a significant health concern [4]. While effective vaccines have been developed for other flaviviruses, including yellow fever and Japanese encephalitis, there is currently no approved human vaccine for WNV. As such, a more comprehensive understanding of immune responses elicited by the virus, including the aspects of these responses that accompany favorable and unfavorable outcomes, is a highly desirable goal.

Although it is clear that both innate immunity and multiple components of the adaptive response play a role in WNV clearance, direct and indirect evidence indicates that CD4+ T cells play a crucial role in protection against WNV infection. For example, Brien et al. [5] demonstrated that WNV-specific CD4+ T cells are sufficient for protection from WNV in a murine viral challenge model and exhibit direct cytotoxic activity. In particular, CD4+ T cell responses have been shown to be essential for clearance of WNV from the central nervous system (CNS) of infected mice [6]. Indirect evidence from human studies also supports the importance of CD4+ T cell responses. For example, WNV infected donors with neurological symptoms were shown to exhibit higher frequencies of CD4+ T cells that expressed Tim-3, which acts as a negative regulator of Th1 cytokine secretion by T cells [7]. This observation implies that the functional characteristics of WNV-specific T cell responses can differ between subjects with neuroinvasive versus asymptomatic infection and that the quality of T cell responses may influence outcomes.

Although T cell responses appear to play an essential role in protecting the host from WNV infection, emerging evidence also suggests that over-exuberant T cell responses may play a role in the pathology of WNV infection. For example, some WNV patients present with autoimmune neuromuscular diseases and demyelinating neuropathies including Guillain-Barre syndrome, myasthenia gravis and stiff-person syndrome, suggesting that immune activation by WNV antigens can lead to immune mediated neurologic damage and/or subsequent cross-recognition of self-antigens [8]. Interestingly, mice that lack perforin and the FAS/FASL pathway were shown to be protected against encephalitis, suggesting that immune mediated damage through both of these pathways may be responsible for neurologic inflammation [9]. More recently, subjects with symptomatic WNV infection were shown to have decreased frequencies of regulatory T cells, implying that regulatory T cells (Tregs) may play a role in limiting WNV disease by restricting pathogenic aspects of the immune response [10, 11].

Cumulatively, these findings suggest that while antiviral T cell immunity is crucial for viral clearance, immune damage mediated by over-exuberant responses may be a component of WNV induced neuropathology. Therefore in this study we sought to characterize WNV-specific T cell responses in subjects who had recovered from laboratory diagnosed WNV infection, including seropositive blood donors with asymptomatic infections and subjects with evidence of neuroinvasive disease recruited through public health organizations. Through the use of HLA class II tetramers, we defined an array of epitopes derived from the structural and non-structural proteins of WNV that are recognized by CD4+ T cells. Utilizing the corresponding tetramers, we performed direct *ex vivo* analysis of CD4^+^ T cell responses in subjects with asymptomatic or neuroinvasive infections, examining the number and phenotype of WNV-epitope specific T cells. We further assessed the phenotype of responses in these subjects to correlate the induced T cell response with clinical outcome following WNV infection. This characterization also included assessment of the cytokine profiles of WNV specific T cells and preliminary transcript profiling of WNV-specific responses. Our results demonstrate that the absolute number of WNV specific CD4^+^ T cells was higher in subjects with neuroinvasive WNV infection, regardless of their HLA restriction or antigen specificity. WNV specific CD4^+^ T cells from subjects with neuroinvasive infection had increased cytokine production and in these subjects a distinct transcript profile was up regulated in response to WNV-specific stimulation. We then examined the frequency and phenotypic profile of CD4+FoxP3 positive T cells to investigate the possible role of Tregs in restraining WNV-specific responses. Our findings implicate a phenotype of increased immune activation in subjects with neuroinvasive WNV infection that is partially explained by a deficit in immune regulation.

## Results

### Study population

Subjects with prior WNV infection were recruited through the Benaroya Research Institute Infectious Disease Repository and through the Blood Systems Research Institute, with written consent as part of IRB approved studies. Subjects with asymptomatic or mild infections were primarily identified through routine WNV screening of United Blood Services blood donations [10]. Subjects with neuroinvasive infections were identified through Washington and Arizona State and county healthcare organizations. All subjects classified as having neuroinvasive disease had an illness with evidence of an acute infectious process such as fever along with clinical evidence of CNS infection, whereas subjects with asymptomatic or mild infections presented for blood donations with either no symptoms or minimal nonspecific symptoms, such as low grade fever or malaise. The attributes of these subjects are summarized in **Table 1**. A total of 24 subjects (12 male and 12 female) with asymptomatic infection were recruited; these subjects had an age range of 23–68 years of age (median age = 53, average age = 51) and were sampled 59–765 days after diagnosis (median duration = 486 days, average duration = 397 days). A total of 16 subjects (8 male and 8 female) with neuroinvasive infection were recruited; these subjects had an age range of 32–85 years of age (median age = 61, average age = 56) and were sampled 299–742 days after diagnosis (median duration = 622 days, average duration = 582 days). Comparing the asymptomatic and neuroinvasive subject groups, there was no significant difference in age (p = 0.21) but the neuroinvasive cohort had a slightly higher proportion of subjects who were over 65 years of age (25% versus 17.4%). The duration that asymptomatic group was sampled after diagnosis was significantly shorter (p = 0.016) than the neuroinvasive group, mainly due to five subjects who were sampled less than 90 days (earliest sampling was at 59 days) after diagnosis.

**Table 1:** Characteristics of WNV infected subjects.

Group^*^	Age	Sample Time^@^	Gender	HLA-DRB1 Type^#^	HLA-DRB1 Type^#^	Assays^^^
**Asymp #1**	59	730	Male	03:01	04:04	A, E
**Asymp #2**	60	765	Female	01:01	04:01	A, E
**Asymp #3**	38	547	Female	08:01	01:01	A, E
**Asymp #4**	67	486	Female	04:01	15:01	A, E
**Asymp #5**	52	612	Female	04:01	13:01	A, E
**Asymp #6**	58	364	Male	04:04	15:01	A, E
**Asymp #7**	53	192	Female	04:01	13:01	A, E
**Asymp #8**	41	602	Female	01:01	13:05	A, B, E
**Asymp #9**	32	707	Female	01:01	04:01	A, B, C, E
**Asymp #10**	64	627	Male	07—	01:01	A, B, E
**Asymp #11**	54	662	Female	04:01	08:01	A, B, C, E
**Asymp #12**	68	564	Female	03:01	07:01	B, E
**Asymp #13**	55	594	Female	01:01	11:04	B, D, E
**Asymp #14**	35	65	Male	07:01	13:02	B, C, E
**Asymp #15**	67	104	Male	03:01	03:01	B, C, E
**Asymp #16**	42	608	Female	01:01	13:05	C, D, E
**Asymp #17**	61	62	Male	04:08	15:01	C, D, E
**Asymp #18**	68	71	Male	01:01	04:03	C, D, E
**Asymp #19**	44	64	Male	04:01	10—	C, D, E
**Asymp #20**	30	59	Male	07:01	13:02	C, D, E
**Asymp #21**	23	123	Male	07:01	08:01	C, D, E
**Asymp #22**	36	176	Male	03:01	15:01	C, E
**Asymp #23**	52	433	Female	13—	15—	C, E
**Asymp #24**	55	314	Male	07—	04:02	D, E
**Average**	50.6	397	**M/F =** 12/12			
**Neuro #1**	66	682	Female	01:01	09:01	A, D, E
**Neuro #2**	85	565	Female	04:01	11:04	A
**Neuro #3**	38	575	Male	04:04	04:04	A, D, E
**Neuro #4**	79	381	Male	04:01	13:01	A
**Neuro #5**	52	663	Male	04:04	07—	A, E
**Neuro #6**	63	481	Male	04:01	03:01	A, C, E
**Neuro #7**	40	711	Female	04:01	13:01	A, C, D, E
**Neuro #8**	41	299	Female	01:01	11:01	A, B, C, D, E
**Neuro #9**	61	735	Female	01:01	03:01	A, B, C, D, E
**Neuro #10**	70	607	Male	04:01	13:02	A, B, C, E
**Neuro #11**	45	656	Female	04:01	07:01	A, B, C, D, E
**Neuro #12**	65	594	Male	03:01	07—	B, D, E
**Neuro #13**	61	636	Female	04:07	03:01	B, E
**Neuro #14**	61	742	Male	07—	04:02	B, C, E
**Neuro #15**	32	656	Male	07:01	12—	B, C, D, E
**Neuro #16**	43	333	Female	07:01	15:01	C, D, E
**Average**	56.4	582	**M/F =** 8/8			

* Asymptomatic subjects had either no symptoms or mild non-specific symptoms. Subjects with neuroinvasive infection had neurologic symptoms, including confusion, tremors, seizures, neck stiffness, paralysis, numbness, vision loss, vertigo, dysarthria, or dysphagia.

^@^ Sample time indicates the number of days elapsed between diagnosis and sampling

^#^ Dashes indicate subjects for whom the high resolution HLA typing result was inconclusive

^ A indicates subjects sampled for *ex vivo* tetramer staining, B intracellular cytokine staining, C transcript profiling, D Treg analysis, and E indicates subjects who were utilized to compare neuroinvasive versus asymptomatic WNV.

### WNV specific T cells recognize a broad range of epitopes

WNV, like other arboviruses in the *Flaviridae* family, contains a single-stranded RNA genome that encodes the capsid (C), envelope (E), premembrane (prM), and membrane (M) proteins, as well as seven nonstructural proteins (NS1, NS2a, NS2b, NS3, NS4a, NS4b, and NS5) that likely contribute to viral replication [12]. We utilized a tetramer guided approach (**S1 Fig**) to comprehensively identify CD4^+^ T cells epitopes across the entire WNV proteome, utilizing peripheral blood samples from subjects with documented WNV infection (**Table 1**). These epitope defining experiments focused on DRB5*01:01 and nine common HLA-DRB1 alleles that cumulatively provided complete coverage for our cohort of WNV infected subjects. As summarized in **Table 2**, this approach allowed us to identify a total of 152 HLA/peptide combinations (from a library of 451 overlapping peptides that represents the entire WNV proteome) that were immunogenic in WNV infected subjects. A complete listing of these epitopes is provided in **S1 Table**. CD4+ T cell epitopes were present in every WNV protein, but only the envelope protein had epitopes that were presented by all of the HLA that we studied (Table **2**). WNV membrane and envelope had the highest epitope densities (8.0 and 7.2 epitopes per 100 amino acids, respectively), while NS5 (the largest protein) contained the highest overall number of epitopes (43 epitopes). Capsid, which is the smallest WNV protein, also had a high epitope density (6.6 epitopes per 100 amino acids), containing 7 epitopes despite its modest size. In contrast, NS2a, NS2b and NS4a proteins were less immunogenic, containing only two epitopes each (**Table 2**). Cumulatively these results show that although WNV specific CD4+ T cells recognize a broad range of epitopes, the response focuses on a subset of structural and non-structural proteins.

**Table 2:** Density of WNV protein epitopes among ten HLA-DR alleles.

HLA (# of subjects)	C [122]	PrM [102]	M [75]	Env [501]	NS1 [353]	NS2a [234]	NS2b [131]	NS3 [619]	NS4a [149]	NS4b [255]	NS5 [905]	Total [3446]
DRB1*15:01 (4)	0	0	1	2	0	1	0	4	0	0	2	10
DRB1*11:04 (3)	2	0	0	3	3	0	0	0	0	2	8	18
DRB1*04:04 (5)	0	0	0	3	0	0	1	5	0	2	8	19
DRB1*04:01 (7)	0	0	0	7	2	0	0	1	0	0	4	14
DRB1*03:01 (4)	0	0	2	1	0	0	0	5	0	1	2	11
DRB1*01:01 (4)	1	0	1	7	2	0	1	4	1	1	5	23
DRB1*01:03 (2)	1	0	0	1	2	1	0	5	0	0	5	15
DRB1*07:01 (4)	0	0	2	3	0	0	0	0	0	1	0	6
DRB1*11:01 (2)	2	0	0	6	5	0	0	2	0	0	5	20
DRB5*01:01 (5)	2	0	0	3	1	1	0	1	1	3	4	16
Total	8	0	6	**36**	15	3	2	**27**	2	10	**43**	152
Density^*^	**6.6**	0	**8.0**	**7.2**	4.2	1.3	1.5	4.4	1.3	3.9	4.8	4.4

^*^ Epitope density for each WNV protein was calculated as the total number of epitopes per 100 amino acids in the corresponding protein. Numbers indicating the highest total numbers of epitope or highest epitope densities appear in boldface.

### WNV specific T cell responses have variable magnitude and exhibit a Th1 memory phenotype

**Fig 1:**
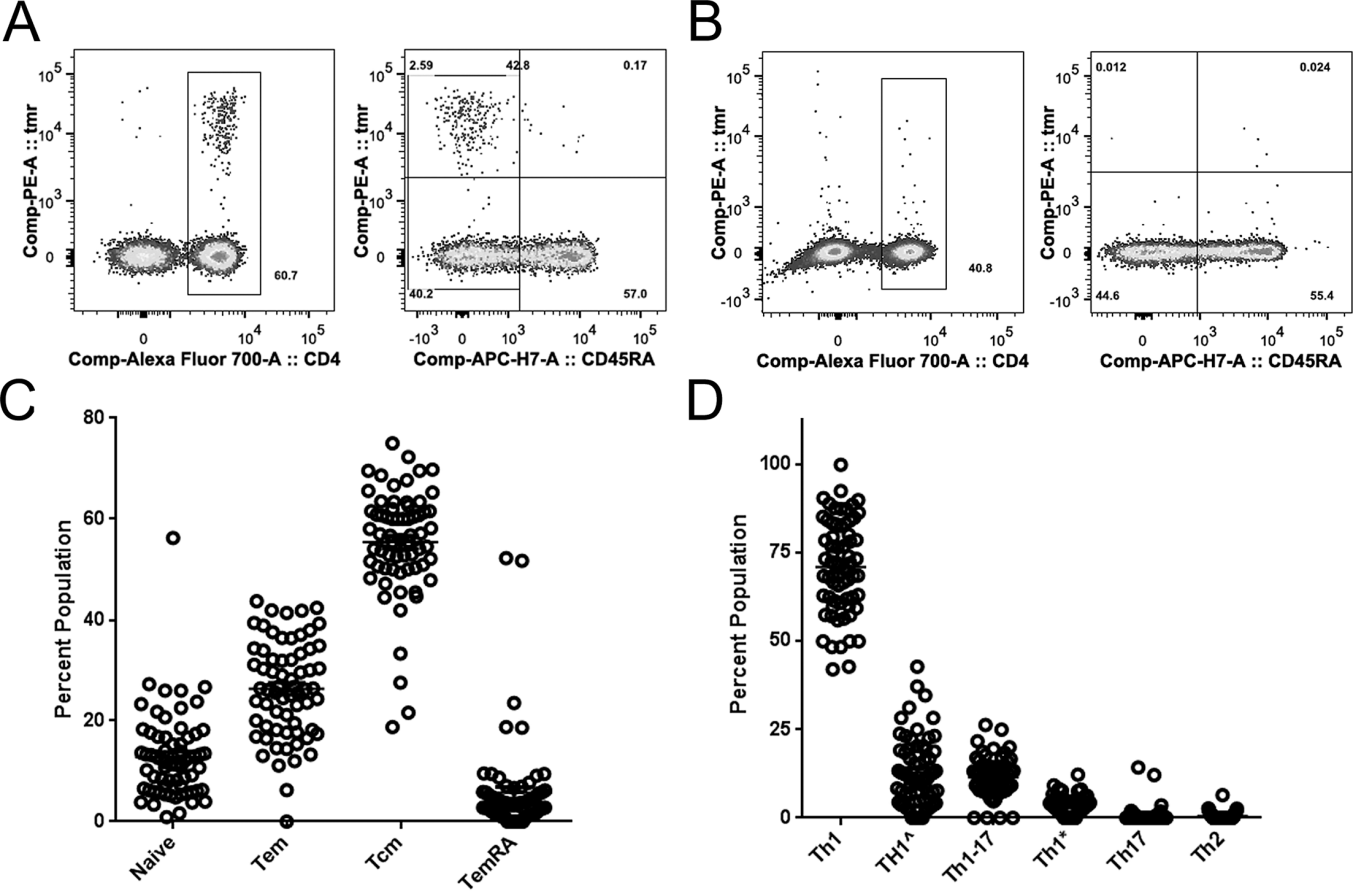
Enumeration and phenotypic characterization of WNV-specific T cells. A) Direct ex vivo tetramer staining of DRB1*01:01/NS1 205–220 specific T cells in 30 million peripheral blood mononuclear cells (PBMC) from a representative WNV infected subject. This tetramer labeled a clear population of CD4+ T cells (left panel) that were predominantly CD45RA- (right panel). B) Direct ex vivo tetramer staining of DRB1*01:01/NS1 205–220 specific T cells in 90 million PBMC from an HLA matched control subject. This tetramer labeled a diffuse population of CD4+ T cells (left panel) that were predominantly CD45RA+ (right panel). C) Summary of naïve and memory T cell phenotypes for WNV infected subjects based on ex vivo surface staining of CD45RA and CCR7. D) Functional heterogeneity of memory T cell phenotypes for WNV infected subjects based on ex vivo surface expression of various combinations of CXCR3, CCR4, and CCR6 on CD45RA- cells. For A-D, T cells specific for single WNV protein epitopes were co-stained with PE-labeled tetramers and CD45RA, CXCR3, CCR4, CCR6, CCR7, and CD38 antibodies. Each data point represents the percent expression of the appropriate combination of surface markers by T cells stained with a single tetramer from a single subject. Staining was performed for a total of 22 subjects using 3 tetramers per subject (66 total stains).

To further characterize WNV-specific responses in previously infected subjects, we used phycoerythrin (PE) labeled tetramers corresponding to a selected set of epitopes to detect WNV-specific CD4^+^ T cells directly *ex vivo* after magnetic enrichment. The patients from our cohort of WNV infected included a total of 26 subjects (15 classified as being asymptomatic and 11 classified as having neuroinvasive infections) who had DRB1*01:01, DRB1*04:01, or DRB1*04:04 haplotypes (**Table 1)**. Tetramers were chosen for these HLA to include at least one envelope epitope and another epitope from at least one non-structural protein (Env 127–144, Env 246–263, and NS1 205–220 for DRB1*01:01; Env 164–180, Env 356–373, and NS1 205–220 for DRB1*04:01; Env 246–263, NS3 91–107 and NS5 159–174 for DRB1*04:04) and a total of 23 subjects (11 of these were classified as being asymptomatic and 11 of these were classified as having neuroinvasive infections) were selected for direct *ex vivo* tetramer analysis. Asymptomatic subject #9 was heterozygous for DRB1*01:01 and DRB1*04:01, and therefore was analyzed with both sets of tetramers. More comprehensive experiments were not feasible because of the large number of cells and technical demands of direct *ex vivo* tetramer analysis. As depicted by the representative *ex vivo* staining result in **Fig 1A**, we consistently detected a distinct population of CD4^+^ memory T cells specific for WNV epitopes using these tetramers. In contrast, when tetramers were used to detect WNV-specific CD4^+^ T cells in subjects with negative WNV serology and no known history of WNV exposure (**Fig 1B**), these subjects had low numbers of WNV-specific CD4^+^ T cells that exhibited a naïve (CD45RA+CCR7+) phenotype. In spite of the fact that subjects were sampled 8–27 months after infection (average time after diagnosis was 18 months), WNV-specific T cells were present at high overall numbers in some subjects. Although we were keenly interested in examining differences between subjects with asymptomatic versus neuroinvasive infections, we first characterized the general attributes of WNV specific T cell response in all subjects. As shown in **S2 Fig**, the number of WNV specific T cells differed for epitopes derived from different antigens (**S2 Fig** panel A) but did not vary significantly based on HLA-DR restriction (**S2 Fig** panel B). In general, we observed significantly higher numbers of T cells specific for envelope and NS1 epitopes than T cells specific for NS3 and NS5 epitopes (although NS3 and NS5 were only evaluated in subjects with DRB1*04:04 haplotypes).

As shown in **Fig 1C**, expression or lack of CD45RA and CDR7 expression by WNV specific T cells indicated that these cells have a predominantly central memory phenotype (representative staining results for single markers are shown in **S2 Fig** panel C). However, some subjects also had a significant proportion of WNV specific T cells that exhibited an effector memory phenotype. For WNV specific memory T cells (CD45RA- cells that were either CCR7+ or CCR7-) we assessed co-expression of various well-defined combinations of lineage associated surface markers to draw inferences about the functional heterogeneity of WNV specific CD4+ T cell responses. These Th subsets were defined essentially as described by Becattini et al. [13], including Th1 (CXCR3^+^CCR4^-^CCR6^-^), Th2 (CCR4^+^CXCR3^-^CCR6^-^), Th17 (CCR6^+^CCR4^+^CXCR3^-^), Th1-17 (CXCR3^+^CCR4^-^CCR6^+^) and non-conventional Th1* (CCR6^+^CXCR3^+^CCR4^+^) subsets–plus a newly defined Th1^ (CXCR3^+^CCR4^+^CCR6^-^) subset. As shown in **Fig 1D**, the relative hierarchy for these different Th subsets was: Th1 > Th1^ > Th1-17 > Th1* > Th17 > Th2. Beyond this, some subjects had a significant proportion of WNV specific T cells that were CD38+ (**S2 Fig** panel C), which is typically indicative of recent activation when expressed on memory T cells. It seemed possible that WNV specific T cells could be activated and maintained in some subjects by the presence of antigen due to persistent virus. Therefore, we examined blood and urine samples from our cohort, testing for the presence of WNV RNA using transcription-mediated amplification (TMA) assays. However, we were unable to detect any WNV RNA in either sample.

**Fig 2:**
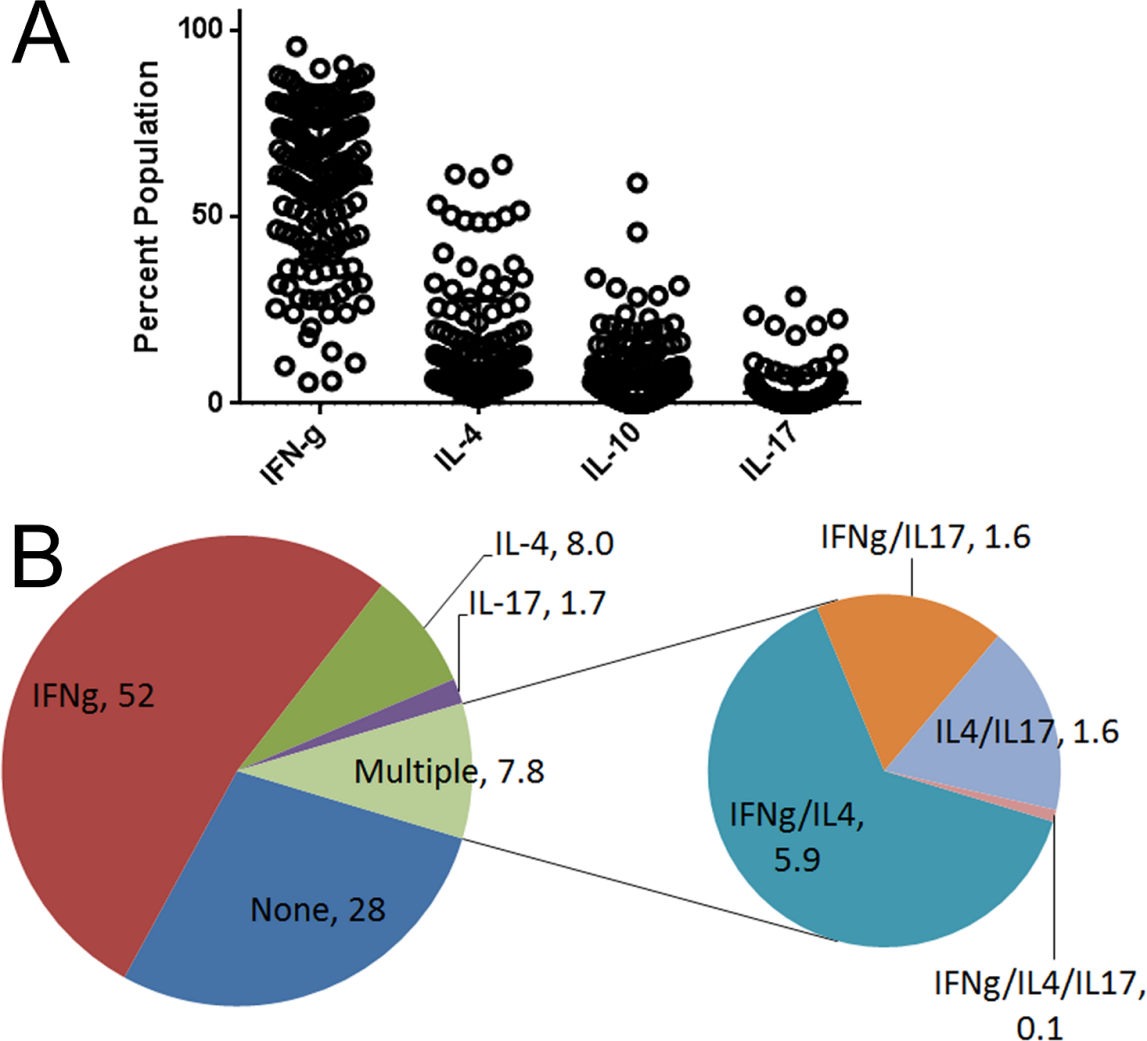
Cytokine profiles of WNV-specific T cells A) WNV-specific T cell lines specific for single epitopes were isolated from WNV infected subjects and analyzed for IFN-γ, IL-4, IL-10, and IL-17 content by intracellular cytokine staining. Each symbol indicates the percentage tetramer positive T cells that were cytokine positive within a single cell line. B) Averaging across all subjects tested, the large pie charts depicts the proportion of WNV specific T cells in subjects with WNV infections that stained positive for IFN-γ only, IL-4 only, IL-10 only and IL-17 only, or any combination of these cytokines ('multiple'). Within the 7.8% of T cells that produced multiple cytokines, the small pie charts indicate the proportion of cells that stained positive for IFN-γ and IL-4; IFN-γ and IL-17; IL-4 and IL-17; or IFN-γ, IL-4, and IL-17. For all other possible combinations the percentage observed was negligible. These data were obtained from a total of 144 WNV specific lines isolated from 16 different subjects.

To follow up on these observations, we analyzed the intracellular cytokine profiles of memory T cells following one round of *in vitro* expansion, using unbiased culture conditions. As indicated in Table 1, these experiments utilized samples from 8 subjects with asymptomatic infections (4 of which were also sampled for *ex vivo* tetramer analysis) and 8 subjects with neuroinvasive infections (4 of which were also sampled for *ex vivo* tetramer analysis). Multiple cell lines were isolated from each subject (using a minimum of three different epitopes per subject) resulting in a total of 144 WNV specific lines (81 from neuroinvasive subjects and 63 from asymptomatic subjects). In these experiments, CD4+ T cells were co-stained with tetramer and various anti-cytokine antibodies after *in vitro* expansion (representative results are shown in **S3 Fig**). Consistent with the observed *ex vivo* surface phenotype, interferon γ (IFN-γ) was the predominant cytokine produced by WNV specific T cells (**Fig 2A**). However, smaller percentages of WNV specific T cells produced detectable levels of interleukin 4 (IL-4), interleukin 10 (IL-10) or interleukin 17 (IL-17). A minor but notable percentage of WNV specific T cells produced more than one cytokine (**Fig 2B**). Among these dual production of IFN-γ and IL-4 was the most prevalent combination. To draw inferences about the relationship between the *ex vivo* surface phenotype of WNV specific T cells and the functional profiles of WNV specific T cell lines, we compared the surface expression of various combinations of CXCR3, CCR4, and CCR6 on WNV specific T cells with the intracellular cytokine profiles of WNV specific T cell lines obtained from the same subjects. Regression analysis (shown in **S4 Fig**) indicated a modest correlation between the percentage of T cells with an *ex vivo* Th2 (CCR4+CXCR3-CCR6-) surface phenotype and IL-4 production by WNV specific T cell lines (p = 0.04) and a more significant correlation between the percentage of T cells with an *ex vivo* Th1^ (CXCR3+CCR4+CCR6-) surface phenotype and co-production of IFN-γ and IL-4 by WNV specific T cell lines (p = 0.002).

### Age related variation of WNV-specific T cell responses

**Fig 3:**
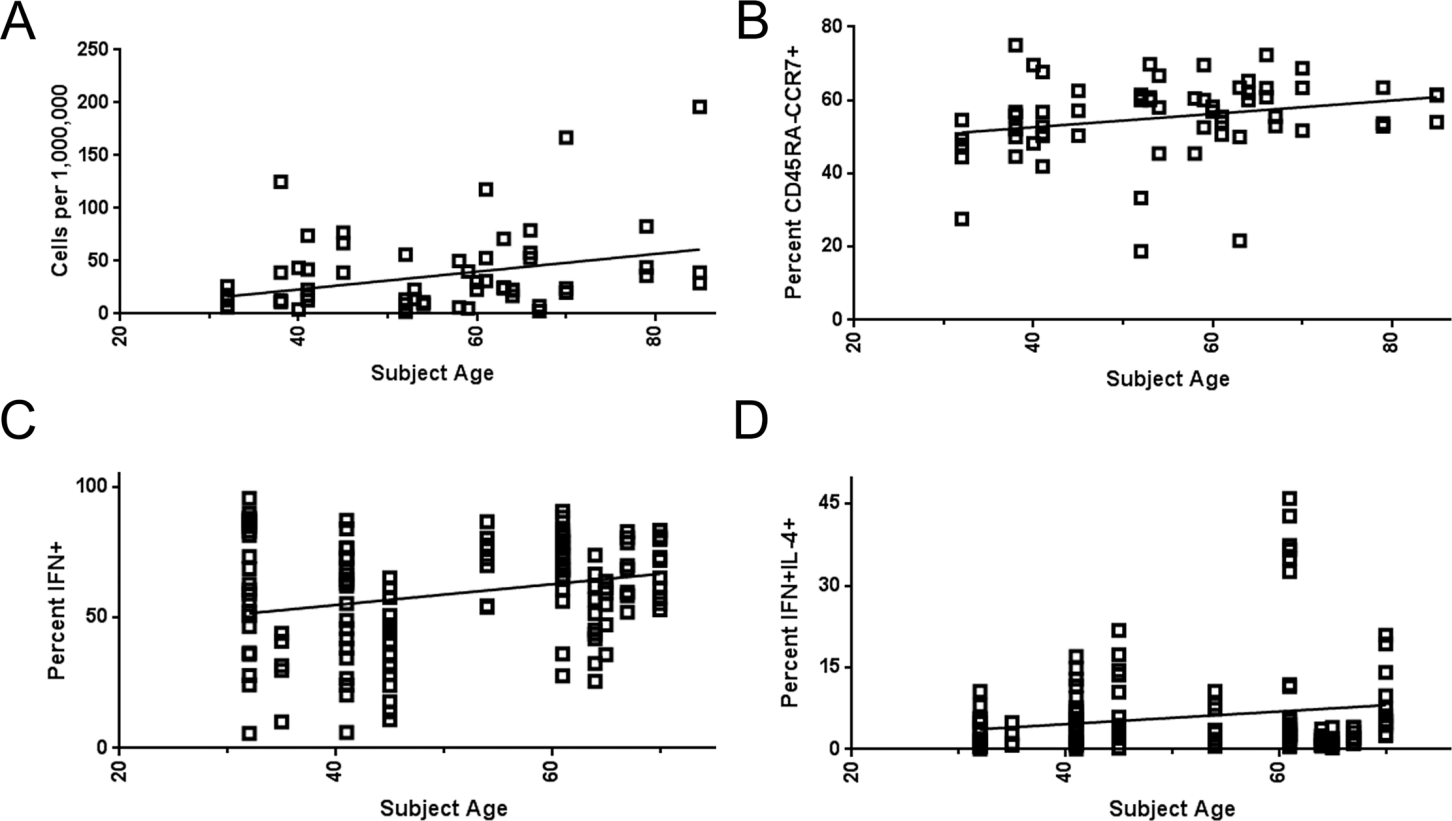
Age related variation in WNV-specific T cell number and function A) Linear regression analysis of WNV-specific T cell number (enumerated by ex vivo tetramer staining for 22 different subjects) versus subject age yielded a positive slope that was significantly non-zero (p = 0.0086) indicating that older subjects tended to have more WNV-specific T cells. B) Linear regression analysis of the percentage of WNV-specific T cells that were CD45RA-CCR7+ versus subject age yielded a positive slope that was significantly non-zero (p = 0.049) indicating that older subjects tended to have a higher proportion of WNV-specific T cells that were TCM. C) Linear regression analysis of IFN-γ production by WNV-specific T cell lines (isolated from 16 different subjects) versus subject age yielded a positive slope that was significantly non-zero (p = 0.0021) indicating that older subjects had a higher proportion of WNV-specific T cells that were IFN-γ positive. D) Linear regression analysis of IFN-γ and IL-4 co-production by WNV-specific T cell lines versus subject age yielded a positive slope that was significantly non-zero (p = 0.031) indicating that older subjects tended to have a higher proportion of WNV-specific T cells that were IFN-γ and IL-4 double positive.

Although WNV specific memory T cell responses were detectable in all WNV infected subjects, we observed a wide range of magnitudes and heterogeneous phenotypes for WNV specific T cells. It is well known that elderly subjects have an increased risk of neuroinvasive WNV infection, perhaps due to age related differences in immunity [14], and the overall age distribution of our study population was diverse–ranging from 23 to 85 years of age. Therefore, we performed multiple regression analyses to investigate the influence of age on the magnitude and phenotype of WNV-specific T cell responses. Interestingly, regression analysis indicated that older subjects generally had significantly higher numbers of WNV-specific T cells (p = 0.009) (**Fig 3A**) and tended to have a higher proportion of T cells with a central memory phenotype (p = 0.049) (**Fig 3B**). All other comparisons of surface marker expression, including other naïve and memory subsets (classified based on surface expression of CD45RA and CCR7) and functional subsets (classified based on surface expression of various combinations of CXCR3, CCR4, and CCR6), did not vary significantly versus age. Examining the cytokine profiles of WNV specific T cell lines isolated from various subjects, regression analysis indicated that WNV specific lines isolated from older subjects had a higher proportion of T cells that produced IFN-γ (p = 0.0021) (**Fig 3C**) and a higher proportion of T cells that co-produced IFN-γ and IL-4 (p = 0.032) (**Fig 3D**). It seemed possible that other factors such as variations in HLA type or the time since diagnosis could influence T cell number or function. However, additional regression analysis indicated that there was no significant correlation between WNV specific T cell number or function and HLA type or time since diagnosis.

### Higher numbers of WNV-specific cells and altered T cell phenotype in neuroinvasive WNV

**Fig 4:**
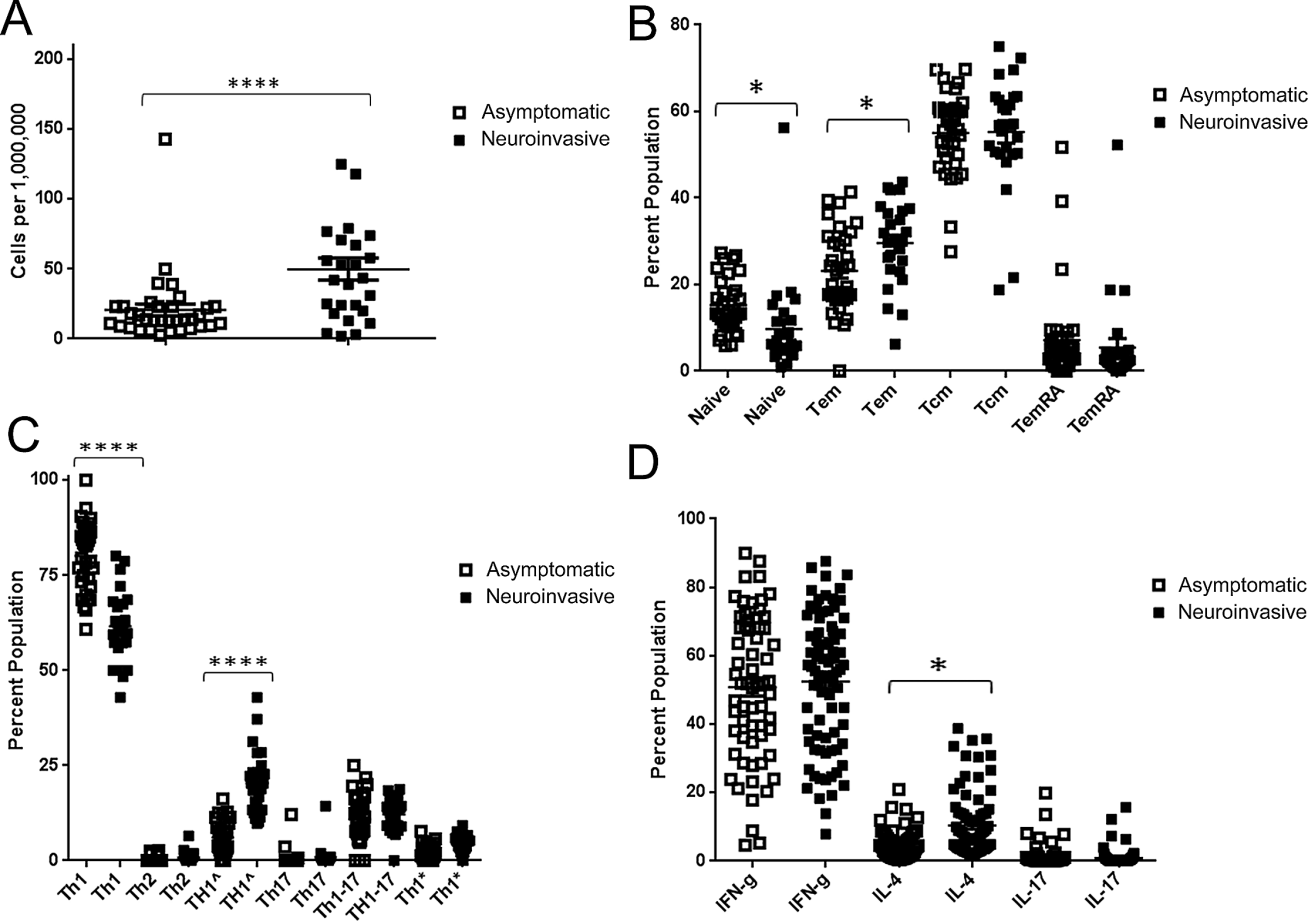
Differences in T cell number and phenotype for subjects with asymptomatic or neuroinvasive WNV infection. A) WNV-specific T cells from a total of 11 asymptomatic subjects (open symbols) and 9 subjects with neuroinvasive infection (filled symbols) as enumerated by ex vivo tetramer staining. Each data point represents tetramer staining for a single WNV epitope (2–3 epitopes were measured per subject) measured in a single subject. Subjects with neuroinvasive infection has significantly more WNV specific T cells (p<0.0001). B) Comparison of the relative proportion of naïve (CD45RA+CCR7+), TEM (CD45RA-CCR7-), TCM (CD45RA-CCR7+), and TEMRA (CD45RA+CCR7-) WNV specific T cells as determined by ex vivo tetramer analysis. Subjects with neuroinvasive infection (filled symbols) had a significantly lower proportion of naïve WNV specific T cells than asymptomatic subjects (open symbols) (p < 0.05) and a significantly higher proportion of TEM. C) Comparison of the relative proportion of WNV specific T cells within various defined functional subsets. Subjects with neuroinvasive infection had a significantly lower proportion of WNV specific T cells that were Th1-like (CXCR3+CCR4-CCR6-) (p < 0.0001) than asymptomatic subjects (p < 0.05) and a significantly higher proportion of Th1^ (CXCR3+CCR4+CCR6-) cells (p < 0.0001). D) Comparison of intracellular IFN-γ, IL-4, and IL-17 staining for WNV specific T cell lines from asymptomatic subjects (open symbols) or subjects with neuroinvasive infection (filled symbols). A significantly higher percentage of WNV specific T cells from subjects with neuroinvasive infections were positive for IL-4 (p<0.05). All other cytokines were not significantly different.

Based on a previously observed association between symptomatic WNV infection and reduced Tregs [10], subjects with neuroinvasive disease could be expected to have stronger WNV-specific CD4+ T cell responses than those with asymptomatic infection. To address this question we directly compared the number of WNV-specific T cells in subjects with asymptomatic infection versus those with neuroinvasive disease. To ensure that this analysis was not confounded by age, we selected groups of subjects (indicated in Table 1) such that the age distributions for the neuroinvasive and asymptomatic groups were statistically indistinguishable (Kolmogorov-Smirnov test, p = 0.639). Consistent with our expectations, subjects with neuroinvasive infection had significantly higher numbers of WNV-specific CD4+ T cells than asymptomatic subjects (**Fig 4A**), averaging 50±4 cells per million (mean ±SEM) and 21±3 cells per million respectively (p < 0.0001). As shown in **Fig 4B**, neuroinvasive subjects had a lower percentage (based on CD45RA and CDR7 expression) of naïve WNV specific T cells (p < 0.05) and, correspondingly, a higher percentage of WNV specific T_EM_ cells (p < 0.05). In contrast, the percentages of T_CM_ and T_EMRA_ cells did not differ between the two groups. Comparing the expression of lineage associated surface markers on WNV specific T cells, we observed that subjects with neuroinvasive infections had a significantly lower percentage of Th1-like (CXCR3^+^CCR4^-^CCR6^-^) cells (p < 0.0001) and a higher percentage of Th1^ (CXCR3+CCR4+CCR6-) cells (p < 0.0001) (**Fig 4C**). Other defined combinations of surface markers were not significantly different. Correspondingly, analysis by intracellular cytokine staining indicated that WNV specific T cell lines isolated from subjects with neuroinvasive infections had a significantly higher percentage of WNV specific T cells that were IL-4 positive (**Fig 4D**). All other cytokines tested were not significantly different.

**Fig 5:**
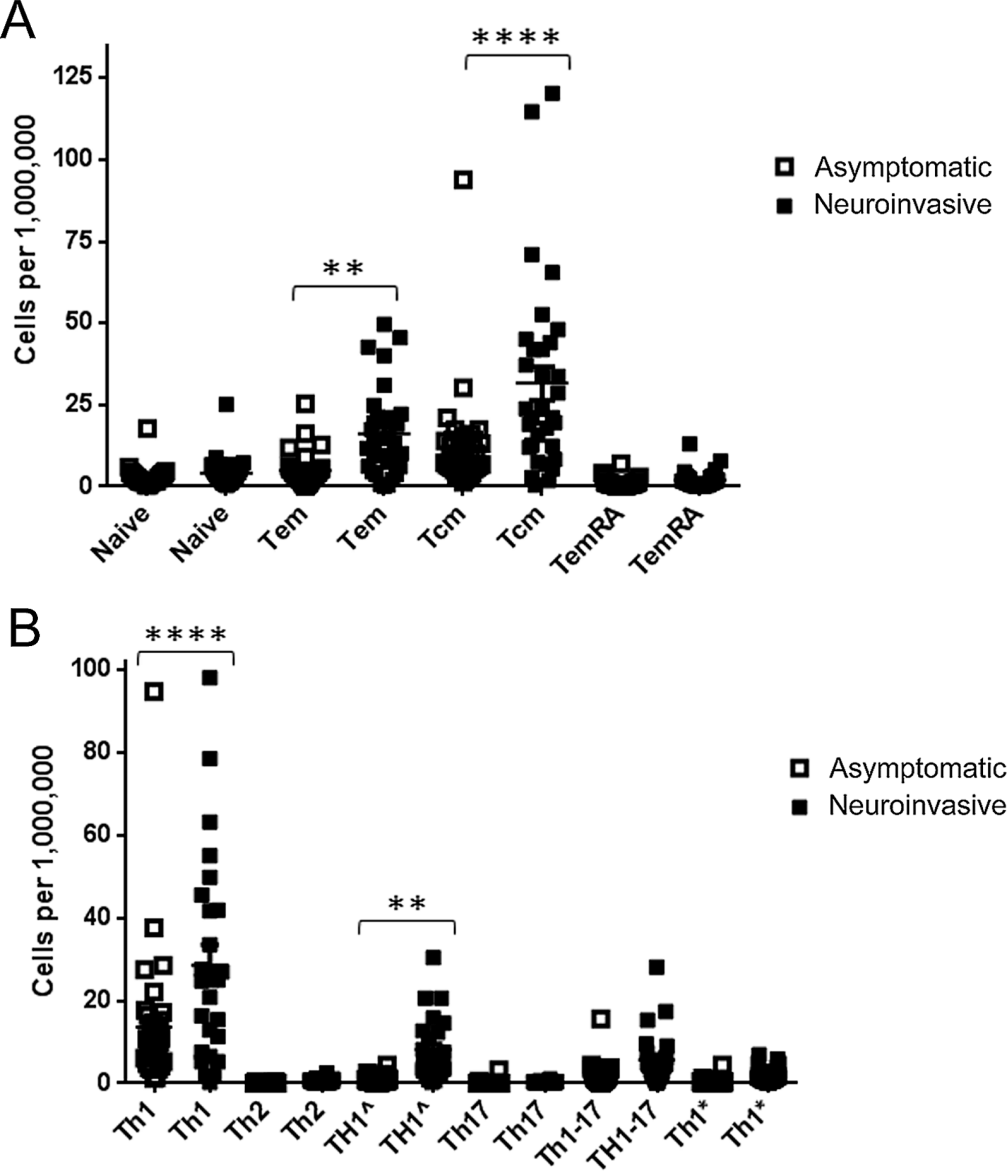
Comparing the absolute number of various subsets of WNV specific T cells in WNV subjects with neuroinvasive versus asymptomatic infection. A) Neuroinvasive subjects (9 subjects, designated by filled symbols) had higher numbers of WNV specific CD4+ T cells that were TEM or TCM (p < 0.0001 and p < 0.01 respectively) than asymptomatic subjects (11 subjects, designated by open symbols). B) Neuroinvasive subjects (filled symbols) had higher numbers of WNV specific CD4+ T cells that were Th1 or Th1^ (p < 0.0001 and p < 0.01 respectively) than asymptomatic subjects (open symbols).

The WNV specific T cell response was ostensibly a Th1 memory response in WNV infected subjects, based on the prevalence of Th1-like (CXCR3^+^CCR4^-^CCR6^-^) T cells and production of IFN-γ as the predominant cytokine by WNV specific T cell lines. However, subjects with neuroinvasive infection were distinguished from asymptomatic subjects by their higher overall number of WNV specific T cells, increased prevalence of T_EM_ cells and reduced prevalence of naïve T cells, increased prevalence of TH1^ (CXCR3^+^CCR4^+^CCR6^-^) T cells and reduced prevalence of Th1 (CXCR3^+^CCR4^-^CCR6^-^) T cells, and increased production of IL-4. To further clarify the quantitative aspects of WNV specific T cell responses in subjects with neuroinvasive versus asymptomatic infections, we next compared the absolute number of T cells within the various T cell subsets that comprise the WNV specific response. As shown in **Fig 5A**, subjects with neuroinvasive disease had increased numbers of WNV specific T_EM_ and T_CM_ (p < 0.01 and p < 0.0001 respectively), whereas both groups had similar numbers of naïve and T_EMRA_ cells. As shown in **Fig 5B**, subjects with neuroinvasive disease had increased numbers of WNV specific Th1 and Th1^ (p < 0.0001 and p < 0.01 respectively), but had similar numbers of all other functional subsets.

**Fig 6:**
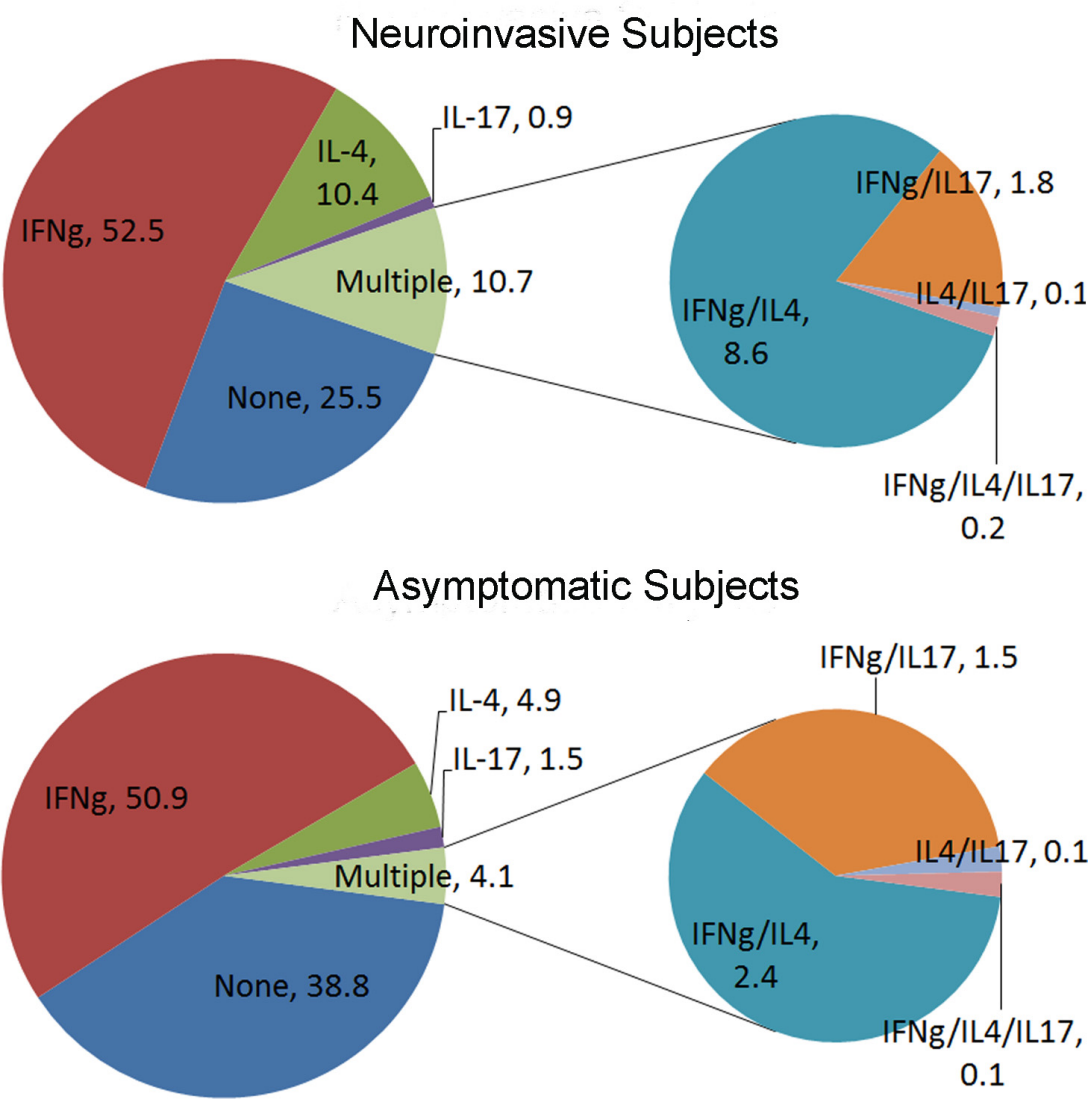
Subjects with neuroinvasive WNV infections have an atypically polarized cytokine response. The large pie charts depict the proportion of WNV specific T cell lines obtained from 8 subjects with neuroinvasive infections (upper panel) or 8 subjects with asymptomatic infections (lower panel) that stained positive for IFN-γ only, IL-4 only, IL-10 only and IL-17 only, or any combination of these cytokines ('multiple'). Within the 10.7% and 4.1% of T cells respectively that produced multiple cytokines, the small pie charts indicate the proportion of cells in subjects with neuroinvasive infections (upper panel) or asymptomatic infections (lower panel) that stained positive for IFN-γ and IL-4; IFN-γ and IL-17; IL-4 and IL-17; or IFN-γ, IL-4, and IL-17. For all other possible combinations the percentage observed was negligible. A significantly higher percentage (p<0.0001) of WNV-specific T cell lines isolated from subjects with neuroinvasive infections were positive for more than one cytokine than from subjects with asymptomatic infections The elevated percentage of WNV-specific T cells that produced dual cytokines in subjects with neuroinvasive infections was almost exclusively due to T cells that co-produced IFN-γ and IL-4. These cells occurred at a significantly higher proportion (p<0.0001) in subjects with neuroinvasive infections than in subjects with asymptomatic infections whereas the proportion of T cells that co-produced other combinations of cytokines did not differ.

The appearance of CXCR3+CCR4+CCR6- T cells as a distinguishing feature of WNV specific T cell responses is interesting. Although cells that co-express CXCR3 and CCR4 are rarely studied, CD4+ T cells that are CXCR3+CCR4+ comprise approximately 5% of CD45RA- cells in peripheral blood and were previously shown to include cells that simultaneously produce Th1 and Th2 cytokines [15]. More recently Araya et al. [16] reported the induction of T box transcription factor 21 (Tbet) and CXCR3 in CCR4+CD25+CD4+ T cells in the context of HTLV-1 infection, leading to induction of IFN-γ secretion by this cell subset. As such, there is precedent to suggest that CXCR3+CCR4+CCR6- T cells represent a population of T cells with an atypically polarized cytokine response. Therefore, we next sought to verify whether subjects with neuroinvasive infections had a significantly higher percentage of WNV specific T cells that co-produced IFN-γ and IL-4. As summarized in **Fig 6**, subjects with neuroinvasive infection had a significantly higher average percentage of WNV specific T cells that produced multiple cytokines than subjects with asymptomatic infections (10.7% versus 4.1%, p < 0.0001). The majority of ‘multiple cytokine positive’ WNV specific T cells co-produced IFN-γ and IL-4 (8.6% and 2.4% respectively), and this was significantly different between the two groups (p < 0.0001). Smaller fractions co-produced IFN-γ and IL-17 (1.8% and 1.5% respectively); IL-4 and IL-17 (0.1% for both groups); or IFN-γ, IL-4 and IL-17 (0.2% and 0.1% respectively), but none of these combinations were significantly different between the two groups. In summary, subjects with neuroinvasive disease are distinguished from asymptomatic subjects by having increased proportions of WNV specific T cells that are T_EM_ and Th1^ (CXCR3+CCR4+CCR6-), higher overall numbers of WNV specific T cells, and higher numbers of WNV specific T cells that are T_CM_, T_EM_, Th1, and Th1^. Correspondingly, WNV-specific T cell lines isolated from those with neuroinvasive disease exhibited an atypically polarized functional response that included a significantly higher proportion of cells that co-produced IFN-γ and IL-4.

### Subjects with neuroinvasive disease have a distinct up-regulated transcript profile

Our results demonstrated clear differences in the magnitude and functional profile of WNV-specific responses in the two subject groups. We next sought to investigate the transcript profiles elicited by stimulation of PBMC with WNV epitopes in these two groups of subjects. To this end, we activated PBMC from subjects who had asymptomatic or neuroinvasive WNV infection for 20 hours using peptides representing the entire WNV envelope protein, collected RNA, and performed whole genome transcript profiling using RNAseq as described in Materials and Methods. In these experiments the PBMC were stimulated using 20mer peptides because this length of peptide primarily serve to activate a CD4+ T cell response. As shown in **Table 3**, 15 transcripts were found to be differentially expressed (p-values less than 0.05 after multiple testing correction). Fourteen of those 15 transcripts were differentially up-regulated in subjects with neuroinvasive infections.

**Table 3:** Differentially regulated transcripts among subjects with asymptomatic versus neuroinvasive WNV infection.

Gene ID	Fold-change	p-value	corr p value	Description
CTAG2	14.00405	2.01E-15	9.40E-12	cancer-testis antigen
F3	51.34001	1.30E-09	4.06E-06	blood coagulation
BCL6	4.311695	1.79E-07	4.18E-04	immune—cell survival
C10orf10	39.05044	5.24E-06	9.19E-03	ELK1 inducer
CCL19	1567.837	1.03E-05	1.71E-02	immune trafficking
MAPK8IP1	12.31179	1.40E-05	2.19E-02	kinase interacting protein
PPAN	4.443578	1.52E-05	2.25E-02	growth/metabolism
SERPINB7	7782.808	1.65E-05	2.32E-02	serpin peptidase inhibitor
CCL3	26.81405	1.83E-05	2.47E-02	immune—inflammatory chemokine
PSAT1	5.116157	2.77E-05	3.55E-02	growth/metabolism
ADTRP	6.593632	1.46E-07	3.86E-04	blood coagulation
PYCR1	13.6462	3.73E-05	3.88E-02	growth/metabolism
F2RL2	32.22259	3.33E-05	3.88E-02	blood coagulation
FITM1	0.046014	4.12E-05	4.08E-02	growth/metabolism
MMP12	104.5879	4.56E-05	4.27E-02	metallopeptidase

**Fig 7:**
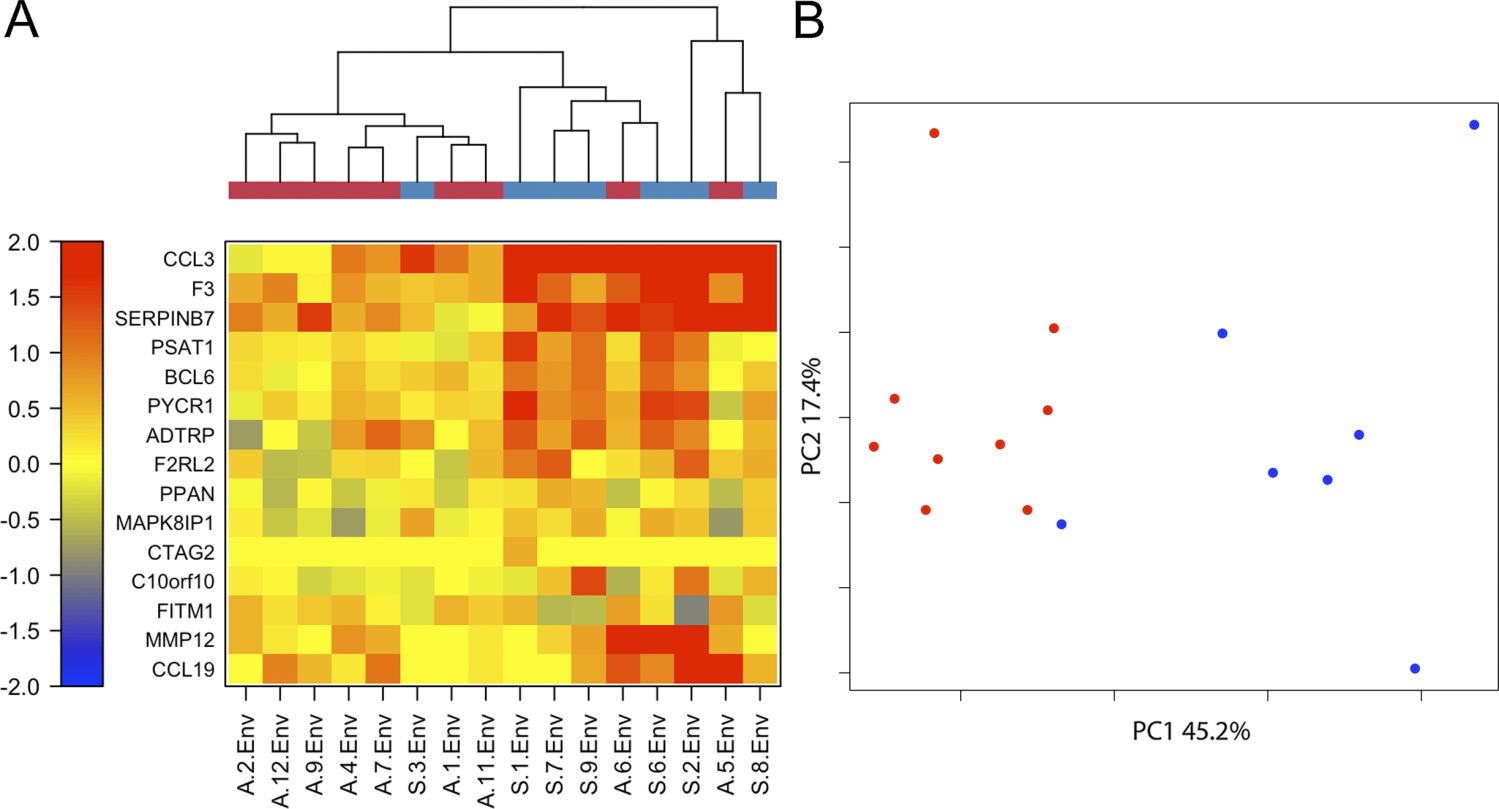
Subjects with neuroinvasive WNV infections have a uniquely upregulated transcript profile. A) Heat map of differentially expressed genes and unsupervised clustering analysis for 7 subjects with neuroinvasive (blue line segments) or 9 subjects with asymptomatic (red line segments) WNV infections. Clusters on the right side of the heat map (which included all but one subject with neuroinvasive infections) had a more up-regulated transcript profiles than the cluster on the left side of the heat map (which included all but two subjects with asymptomatic infections). B) Segregation of subjects with neuroinvasive (blue symbols) or asymptomatic (red symbols) WNV infections based on principal component analysis. This analysis was performed using log2(FC) values for the differentially expressed genes. As indicated on the axes the first two principal components account for 45.2 and 17.4 percent of the variance respectively.

Among these were multiple genes with known or suspected immunological functions (BCL6, CCL19, CCL3, and SERPINB7), genes related to cell growth and metabolism (PPAN, PSAT1, PYCR1, and FITM1), genes related to blood coagulation (F3, ADTRP, F2RL2) and genes from other unrelated pathways (the matrix metallopeptidase MMP12, the ELK1 transcription factor inducer C10orf10, the neuroendocrine related kinase interacting protein MAPK8IP1, and the cancer-testis antigen CTAG2). Notably, a few of these genes have known functions that would be expected to promote anti-viral immune responses. BCL6 is known to promote T cell survival and plays a role in protecting effector T cells from apoptosis [17]. CCL19 is a CCR7 ligand that regulates CD4+ T cell homing and trafficking and also plays a role in initiating signalling [18]. CCL3 (also known as MIP1 alpha) is an inflammatory chemokine that enhances innate immune responses through binding to the CCR1, CCR4 and CCR5 receptors; among these CCR4 was shown to dictate the inflammatory response to TLR challenge [19]. As shown in **Fig 7A**, unsupervised clustering analysis revealed two distinct profiles for these differentially expressed genes: a highly up-regulated profile that included 6 of the 7 subjects with neuroinvasive disease and a more weakly up-regulated profile that included 7 of the 9 asymptomatic subjects. **Fig 7B** depicts a clear segregation of subjects with asymptomatic versus neuroinvasive infections by orthogonal principal components derived from these transcripts. In this analysis, the first two principal components (PC1 and PC2) account for 45.2 and 17.4 percent of the variance respectively. In total, these results indicate that subjects with neuroinvasive infection have a distinct upregulated transcript profile that includes a trio of immunological transcripts (BCL6, CCL19, and CCL3). This would be expected to promote more robust and inflammatory WNV-specific responses through increased survival, activation, homing, and signaling.

### Subjects with neuroinvasive disease have an altered Treg profile

**Fig 8:**
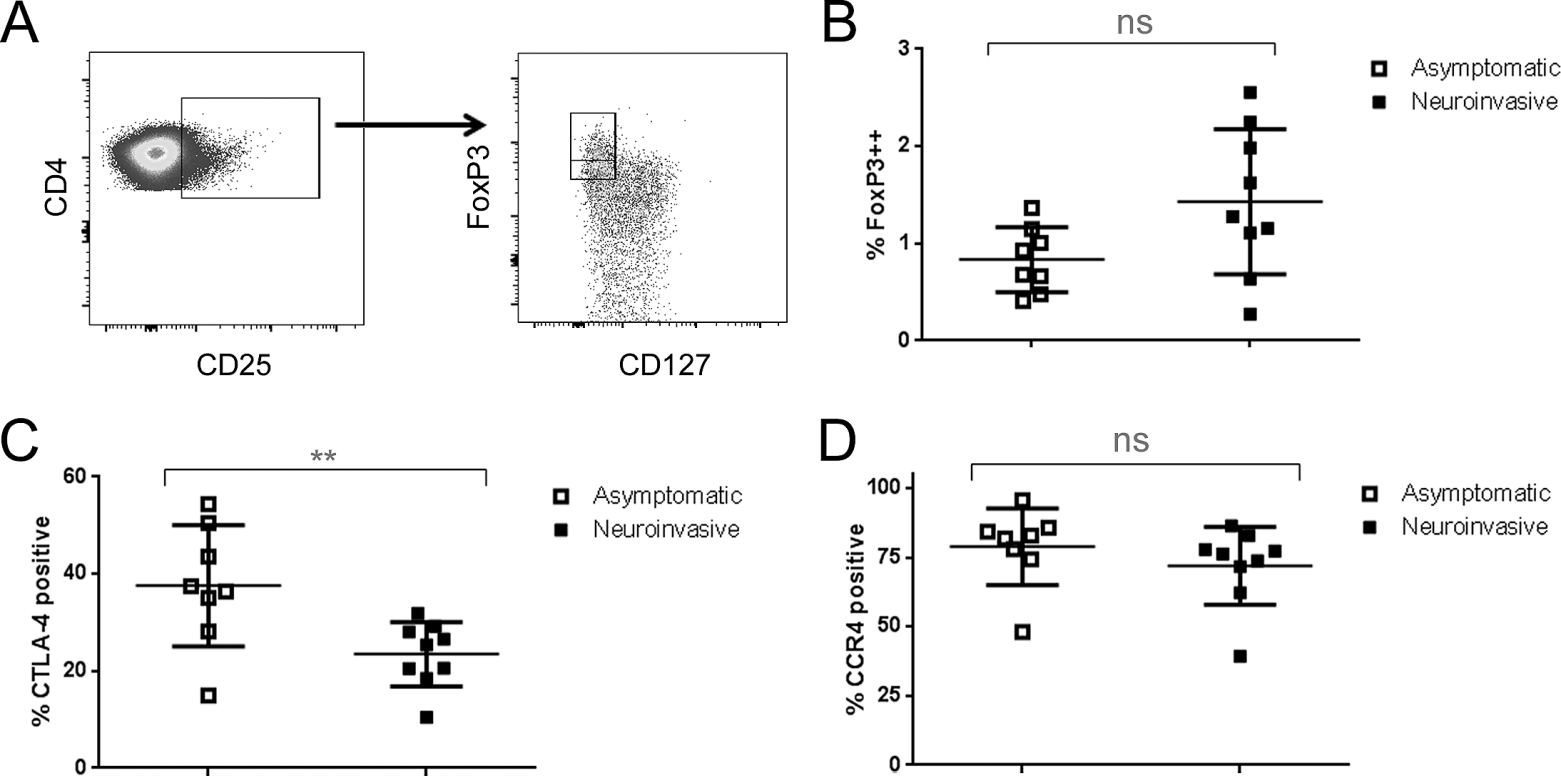
Altered Treg phenotype in subjects with neuroinvasive WNV. A) CD4+ Tregs were evaluated in PBMC samples from 9 subjects with neuroinvasive infection or 8 subjects with asymptomatic WNV by gating on CD25+CD4+ T cells and subsequently gating on CD127lowFoxP3++ T cells (designated by the upper left rectangle). B) Each symbol represents the overall percentage of Treg (CD4+CD25+CD127lowFoxP3++ T cells) within the PBMC of a single WNV infected subject. The percentage of CD4+ Tregs was not significantly different between subjects with neuroinvasive or asymptomatic WNV. C) Each symbol represents the percentage of Treg that were CTLA-4 positive for a single WNV infected subject. Subjects with neuroinvasive WNV had a significantly lower percentage of CTLA-4 positive Tregs (p = 0.0097). D) Each symbol represents the percentage of Treg that were CCR4 positive for a single WNV infected subject. The percentage of Treg that were CCR4+ was not significantly different between subjects with neuroinvasive or asymptomatic WNV.

Prior studies have shown that subjects with symptomatic WNV infection have decreased frequencies of regulatory T cells (10;11). Therefore it is plausible that deficits in regulatory T cell (Treg) number or function could contribute to the formation of an atypical and potentially pathological T cell response in neuroinvasive WNV infection. To investigate this possibility, we utilized a multi-color staining panel to compare the number and phenotype of CD4+Foxp3+ Treg in subjects with neuroinvasive versus asymptomatic WNV infection. As shown in **Fig 8A**, we defined Tregs by gating on CD4+ T cells that were CD25+ and identifying a population that was CD127 low and FoxP3 bright (all of these cells expressed a higher level of FoxP3 than CD127+ cells). Utilizing this gating strategy, we observed no significant difference in the overall percentage of Treg between subjects with neuroinvasive or asymptomatic WNV infection (**Fig 8B**). Due to sampling limitations, we did not have an adequate number of cells available to directly assess Treg function. To draw inferences about Treg quality, we performed stains to characterize the expression of CLTA-4 and CCR4 on Treg, as both of these markers have been reported to correlate with improved suppressive capacity [20]. We observed that subjects with neuroinvasive WNV infection had a much lower proportion of Tregs that were CTLA-4 positive (**Fig 8C**), suggesting a possible defect in Treg function. However, the percentage of Tregs that were CCR4+ was not significantly different between subjects with neuroinvasive or asymptomatic WNV infection (**Fig 8D**).

## Discussion

For reasons that are not fully understood, outcomes for subjects following WNV infection are diverse, raising questions about the mechanisms that influence the severity of disease in infected subjects [21–23]. CD4+ T cell responses have been shown to be sufficient for protection (independent of B cells and CD8 T cells) from WNV challenge and crucial for viral clearance from the CNS, but conflicting evidence suggests that either poor viral clearance or immune mediated tissue damage are important causative factors in neuroinvasive disease [5, 6]. In the present study, we addressed this question by utilizing HLA class II tetramer reagents to directly characterize WNV specific CD4+ T cell responses in subjects with previous WNV infection and compared the immune phenotype in subjects with asymptomatic infections versus neuroinvasive disease. We had previously observed that the yellow fever vaccine elicits T cell responses against every protein in the YFV proteome [24]. Similarly, we observed that CD4+ T cell responses from previously infected subjects with various HLA haplotypes are directed against a wide array of WNV epitopes derived from all of the structural and non-structural proteins. While broad, the epitopes recognized by CD4+ T cells were focused on a subset of viral proteins, including Env, NS1, NS3, and NS5. This observation is in agreement with a previous study in which the majority of positive ELISpot responses in HLA-DR2, HLA-DR3, and HLA-DR4 transgenic mice were directed against peptides from Env, NS3, and NS5 [25].

Utilizing a panel of tetramers, we examined the *ex vivo* characteristics of the WNV-specific CD4^+^ T cell response in subjects with DRB1*01:01, DRB1*04:01, and DRB1*04:04 haplotypes. In general, T cells that recognized Env and NS1 epitopes occurred at higher numbers than those that recognized NS3 and NS5 epitopes, but WNV specific T cells were primarily T_CM_ (CD45RA-CCR7+) and predominantly Th1-like (CXCR3+CCR4-CCR6-) regardless of epitope specificity. Therefore, although the overall number of T cells appears to vary for different WNV antigens, their phenotypic characteristics were similar regardless of epitope specificity. In keeping with their observed surface phenotype, WNV specific T cells mainly produced IFN-γ (and only lesser quantities of IL-4, IL-10, and IL-17), which is consistent with a prior study that observed IFN-γ and IL-2 production by WNV specific T cells following peptide vaccination of mice with CD4+ T cell epitopes [5]. These findings suggest a uniform phenotype for WNV specific T cells that recognize epitopes (from either structural or non-structural proteins with functional characteristics) which likely promote effective antiviral immunity. Examining variations in WNV specific T cell responses with respect to age, we observed that older subjects had a higher number of WNV specific T cells, a greater proportion of which were T_CM_. WNV specific cell lines isolated from older subjects had a higher proportion of T cells that produced IFN-γ and also a higher proportion that co-produced IFN-γ and IL-4. Such responses against WNV were clearly effective in subjects who cleared the virus and remained essentially asymptomatic. Given that age is an established risk factor for neuroinvasive WNV infection, these observations imply that elevated WNV specific T cell responses may promote pathogenic outcomes rather than protection.

We next compared the magnitude and *ex vivo* characteristics of WNV-specific CD4^+^ T cell response in subjects with neuroinvasive disease or asymptomatic infection. For this comparison, we selected groups of subjects with similar age distributions, so that this variable would not confound our analysis. We found that, independent of age subjects with neuroinvasive disease had higher numbers of WNV-specific CD4+ T cells. Among these WNV-specific cells, a lower proportion were naïve and a higher proportion were T_EM_. These differences in proportion coincided with increases in the overall number of T_EM_ and T_CM_ in subjects with neuroinvasive infections. Neuroinvasive disease was significantly associated with an increased proportion and increased number of WNV-specific CD4^+^ T cells that were CXCR3+CCR4+CCR6-. As described by Kim et al. [15], CXCR3 and CCR4 expression have been traditionally used designate T cells as being respectively Th1-like or Th2-like, as 88% of Th1 cells in blood are CXCR3+CCR4-, and 90% of Th2 cells are CXCR3-CCR4+. It has been known for some time that although the majority of memory T cells in the periphery express only one of these markers, approximately 5% are CXCR3+CCR4+ and have the capacity to produce IFN-γ, IL-4, or both [15]. A more recent study by Araya et al. [16] concluded that HLTV-1 infection induced CXCR3 expression in CCR4+CD25+CD4+ T cells, which then produced IFN-γ through viral upregulation of Tbet. The emergence of these cells was apparently correlated with severity of infection. Another recent report [26] described a case of post-transplant adult T-cell leukemia/lymphoma in whom CXCR3+CCR4+ T cells were observed. As recently described by Becattini et al. [13], the repertoire of memory CD4+ T cells includes not only cells polarized towards a single fate (conventional Th1, Th2, etc.), but also cells whose progeny have acquired multiple fates (expressing combinations of chemokine receptors and producing measureable levels of the expected cytokines corresponding to these markers). That study utilized surface expression of various combinations of CXCR3, CCR4, and CCR6 to define the following subsets: Th1 (CXCR3^+^CCR4^-^CCR6^-^), Th2 (CCR4^+^CXCR3^-^CCR6^-^), Th17 (CCR6^+^CCR4^+^CXCR3^-^), Th1-17 (CXCR3^+^CCR4^-^CCR6^+^) and non-conventional Th1* (CCR6^+^CXCR3^+^CCR4^+^). To these we would add Th1^ (CXCR3^+^CCR4^+^CCR6^-^) as a subset that is apparently important in the context of WNV infection. Consistent with coordinate expression of CXCR3 and CCR4, we observed that WNV-specific T cell lines from subjects with neuroinvasive infection had a higher proportion of WNV-specific CD4+ T cells that co-produced IFN-γ and IL-4.

It has been previously demonstrated that subjects with symptomatic WNV infections have decreased numbers of CD4^+^ CD25^+^ Foxp3^+^ Treg cells and mice depleted of Tregs have increased neurological disease and mortality [10]. Therefore, it could be surmised that neuroinvasive infection is likely to be accompanied by over-exuberant immune responses against the virus that are inadequately restrained. Consistent with this notion, we observed that although subjects with neuroinvasive and asymptomatic WNV infections had similar proportions of CD4^+^CD25^+^ CD127^low^Foxp3^++^ cells (implying similar numbers of Treg), Tregs from subjects with neuroinvasive infection had a significantly lower proportion of CTLA-4 expression, which has been shown to be intimately linked to effective suppressive function [20]. Interestingly, Treg ablated mice have also been reported to have significantly higher frequencies of IFN-γ producing CD4+ and CD8+ T cells and an increased proportion of effector T cells that co-produced IFN-γ and TNF-α in response to WNV infection [27]. Therefore, elevated numbers of WNV-specific T cells, many of which co-produce IFN-γ and IL-4, could be construed to imply an inadequately restrained antigen driven response.

Recent functional studies of WNV-specific responses in humans are in conflict about the relationship between the production of various cytokines and different outcomes. Qian et al. [28] report an association between reduced serum IL-4 and severe infection (implying a correlation between reduced IL-4 and worse outcomes), but Garcia et al. [29] report an association between increased pro-inflammatory and antiviral cytokines (such as IFN-γ, IL-2, and IL-6) and prolonged fatigue following symptomatic clinical WNV disease (implying a correlation between increased IFN-γ, IL-2, and IL-6 and worse outcomes). Our results are more in agreement with those of Garcia et al. A recent study of CD8+ T cell responses observed that individuals with neuroinvasive WNV infection had WNV-specific CD8+ T cells that were predominately monofunctional, but T cells from subjects with neuroinvasive disease were shown to have significantly increased CD107a, implying greater cytolytic potential [30]. Similarly, a recent report by Gibson et al. [31] observed that reduced polyfunctional CD4 and CD8 T cell responses were associated with persistent circulating CMV DNA in infants, but this study did not measure other cytokines such as IL-4. Our study provides additional evidence to associate increased functional responses with worse outcomes in the context of viral infection.

Persistence of high numbers of WNV-specific T cells many months after initial infection has several possible explanations. Multiple lines of evidence suggest that subjects with neuroinvasive disease might be expected to exhibit deficient immune responses against the virus leading to delayed viral clearance. Elderly and immunosuppressed subjects have been shown to have an increased risk of neuroinvasive WNV infection [14, 32]. Furthermore, one recent study found that genetic risk variants that are thought to cause impaired resistance to viral infection were associated with symptomatic infection [23]. As such, subjects with neuroinvasive infection may have a higher viral load, leading to higher peak T cell numbers that remain elevated at later times. Indeed, WNV RNA has been shown to persist for three months in blood and others have reported the detection of WNV transcripts at much later times after initial infection [33, 34]. Therefore, it is possible that WNV-specific T cells could be maintained at higher numbers in some subjects by persistent antigen due to the systemic presence of virus. Alternatively, subjects with neuroinvasive disease may differ from asymptomatic subjects only in the amount of virus that reaches their CNS. Indeed, WNV-specific responses were shown to persist in the CNS of mice for 4–6 weeks post-inoculation, implying that virus may persist for longer times in the CNS [35]. In our phenotyping experiments, approximately one third of WNV-specific memory T cells were CD38 positive, implying recent exposure to antigen. However, levels of CD38 were not significantly different between subjects with neuroinvasive infection and asymptomatic subjects. Also, we found no detectable virus in either the blood or the urine of any subjects from our cohort. Alternatively, it is possible that WNV-specific T cells could be maintained and activated by homologous self-peptides. Indeed, WNV infection has been associated with the development of autoimmune neuromuscular diseases [8]. Interestingly, neurologic symptoms have been reported to arise long after acute infection, raising the possibility that cross-recognition of neural tissue could be a secondary event [36].

Our study does have limitations. For logistical reasons (mainly related to recruitment) we were unable to characterize WNV infected subjects at times close to initial infection. Therefore, we were not able to assess parameters such as peak viral titer in our subjects or characterize responses during acute infection. Although we saw clear differences between subjects with neuroinvasive versus asymptomatic infections, it is possible that other compelling differences were present at these earlier time points. In addition, our use of tetramer-based assays allowed detailed and direct interrogation of WNV-specific T cells, but consequently limited this analysis to a few key specificities within the responding T cell population. Therefore, although our data appear to suggest that the functional attributes of the WNV response did not vary for different epitope specificities, we cannot completely rule out that possibility. In addition, although our transcript assays used conditions that were designed to primarily elicit a CD4+ T cell response, these assays were performed on unfractionated PBMC; the resulting profiles could be taken to suggest elevated immune responses that go beyond the CD4+ T cell compartment. This is an important question that exceeds the scope of our current study, but it is likely that future studies may demonstrate important differences in CD8+ T cell or antibody responses that occur in the context of neuroinvasive WNV infection. Finally, our study was inherently limited by its focus on peripheral blood. Although it was only feasible for us access peripheral blood, it is probable that important insights could be gleaned through immune profiling of T cells that have infiltrated neural tissues.

Collectively, our results demonstrate important age related differences in WNV-specific immunity and that, independent of this, WNV specific CD4^+^ T cells occur at higher numbers in subjects with neuroinvasive infection than in asymptomatic subjects, regardless of their HLA restriction or antigen specificity. Neuroinvasive disease was associated with the emergence of a population of WNV-specific TH1^ cells that were CXCR3+CCR4+CCR6- and whose presence correlated with co-production of IFN-γ and IL-4. Furthermore, WNV-specific responses in subjects with neuroinvasive disease up-regulated distinct transcripts (including BCL6, CCL19, and CCL3) that would be expected to promote inflammatory WNV-specific responses through increased survival, activation, homing, and signaling. These results implicate a mechanism of increased immune activation in neuroinvasive disease. However, it remains to be determined whether the increased magnitude of CD4^+^ T cell responses plays a direct or indirect role in the clinical outcome of WNV infection.

## Materials and Methods

### HLA typing, confirmation of WNV infection, and blood/urine testing

To facilitate experiments with HLA class II tetramers DNA samples from each subject were HLA typed using Dynal Unitray SSP Kits (Invitrogen). Laboratory diagnosis of WNV infection was established by either a positive serum or cerebrospinal fluid (CSF) anti-WNV IgM serologic or WNV PCR assay. The subjects were administered a clinical questionnaire either through mail or over the phone. This data was supplemented by medical records obtained from patients’ physicians. Research samples from recruited subjects were tested for WNV RNA using a transcription-mediated amplification assay, the Procleix WNV assay (Hologic) as previously described [37].

### Peptides and tetramer reagents

Libraries of overlapping peptides, generated based on the sequences of each protein in the WNV genome, were obtained through the Biodefense and Emerging Infections Research Resources Repository. These libraries consisted of overlapping peptides, typically 18–20 amino acids long with a 12 amino acid overlap (peptide length and overlap was adjusted as needed to avoid terminal cysteine residues). Peptides were dissolved in DMSO at 20 mg/ml and subsequently diluted as needed. Recombinant HLA-DR proteins were generated as described [38]. Briefly, each HLA-DR was purified from the supernatants of transfected insect cells, biotinylated, and dialyzed into 0.1M phosphate buffer. Biotinylated monomer was loaded with 0.2 mg/ml of peptide by incubating at 37°C for 72 hours in the presence of 2.5 mg/ml n-octyl-β-D-glucopyranoside and 1 mM Pefabloc SC protease inhibitor (Sigma-Aldrich, St. Louis, MO) and then conjugated using R-PE streptavidin (Biosource International, Camarillo, CA) at a molar ratio of 8 to 1.

### Tetramer guided epitope mapping

The Tetramer guided Epitope Mapping (TGEM) procedure was performed as previously described [39]. Briefly, PBMC were isolated by Ficoll underlay and CD4^+^ T cells purified by negative selection using an isolation kit (Miltenyi, Auburn, CA). Adherent cells from the CD4^-^ fraction were used as antigen presenting cells by incubating 2×10^6^ cells per well (200 μL volume) in 48 well plates for 1 h and washing. Two million CD4^+^ T cells per well were stimulated with pools of five consecutive peptides. After 14 days, 1×10^5^ cells were then stained with pooled peptide tetramers. Wells which gave positive staining were stained again using each of the corresponding individual tetramers from the positive pool.

### *Ex vivo* epitope-specific CD4+ T cell analysis

Direct *ex vivo* analysis of epitope-specific T cells *ex vivo*, was accomplished as previously described [24, 40]. Briefly, PBMC at a concentration of 150 million/ml were treated with dasatinib for 10 min at 37°C followed by staining with 20 μg/ml PE-labeled tetramers at room temperature for 120 min. Cells were then labeled with anti-PE magnetic beads and enriched using a magnetic column according to the manufacturer’s instructions (Miltenyi Biotec, Auburn, CA). For phenotyping studies, magnetically enriched cells were stained with antibodies against each marker of interest, including CD3 V500 (BD, clone SP34-2), CD4 AF700 (eBioscience, clone OKT4), CD38 allophycocyanin (APC) (eBioscience, clone HIT2), CD45RA APC-H7 (BD, clone HI100), CXCR3 PE-Cy5 (BD, clone 1C6), CCR4 fluorescein (R&D Systems, clone 205410), CCR6 peridinin chlorophyll protein (PerCP)-Cy5.5 (BD, clone 11A9), and CCR7 PE-Cy7 (BD, clone 3D12). The combination of CD14 Pacific Blue (Invitrogen, clone TuK4), CD20 Pacific Blue (Invitrogen, clone H147), and ViViD fixable violet dead cell stain (Molecular Probes) was used to exclude monocytes, B cells and dead cells from the analysis. Data acquisition was performed on a 17-color BD LSR II instrument and analyzed using FlowJo software (Treestar, Ashland, Ore). T cell numbers (expressed as cells per million) were calculated as previously described [24, 40].

### Intracellular cytokine staining

WNV-specific T cells were expanded by stimulating purified CD4^+^CD45RA^-^ cells with selected WNV peptides *in vitro* for 14 days in the presence of autologous adherent cells as antigen presenting cells. These cells were rested for 3 days and then stained with PE-conjugated tetramers for 30 min at 37°C and stimulated with 25 ng/mL PMA and 1 μg/mL ionomycin at 37°C. Brefelden A was then added and the cells incubated for 3–4 hours at 37°C, 5% CO2. For cytokine staining, surface staining was performed first, followed by fixation/permeabilization as per the manufacturer’s protocol (eBioscience). Cells were then co-stained with antibodies for surface markers and cytokines of interest, including CD3 PE-Cy5 (BioLegend, clone HIT3a), CD4 V500 (BD, clone RPA-T4), IFN-γ AF700 (BioLegend, clone 4S.B3), IL-4 fluorescein isothiocyanate (FITC) (eBioscience, clone 8D4-8), IL-10 PE-Cy7 (Biolegend, clone JE53-9D7), and IL-17 APC-Cy7 (Biolegend, clone BL168), or alternatively with CD4 PerCP-Cy5.5 (BD, clone RPA-T4) and IL-17 APC (Biolegend, clone BL168). ViViD fixable violet dead cell stain was then added to remove dead cells from the analysis. After 20 min at room temperature, cells were washed and immediately analyzed by flow cytometry.

### Treg staining analysis

Frozen PBMC from WNV infected subjects were thawed, rested for 30 minutes and then co-stained with antibodies for surface markers and cytokines of interest, including CD4 PerCP (BD, clone RPA-T4), CD25 PE (BioLegend, clone BC96), CD127 AF488 (Biolegend, clone 8019D5), FoxP3 APC (eBioscience, clone PCH101), CTLA-4 PE-CF594 (BD, clone BN13) and CCR4 BV605 (Biolegend, clone L29IH4). After 20 min at 4 degrees, cells were washed and immediately analyzed by flow cytometry.

### RNA sample preparation

To elicit WNV- and influenza-specific transcript profiles (the latter as a positive control for activation), PBMC from WNV infected subjects were activated using peptides spanning the entire WNV envelope protein or the influenza A/new caledonia/20/99 hemagglutinin protein. Briefly, 2 million PBMC per mL were incubated in 14 mL polypropylene round bottom tubes in the presence of peptides at a final concentration of 1 ug/mL (or a DMSO control) for 20 hours at 37°C, 5% CO2. Cells were then washed, pelleted, and resuspended in RNA later according to the manufacturer’s instructions and stored at -80C. Subsequently, cells were thawed, pelleted, re-suspended in RLT buffer and total RNA was isolated using the Qiagen RNeasy kit according to the manufacturer’s instructions.

### RNA-sequencing

Sequencing libraries were constructed from 100 ng of total RNA using TruSeq RNA Sample Preparation Kits v2 (Illumina). Libraries were clustered onto a flowcell, using a cBOT amplification system with a HiSeq SR v3 Cluster Kit (Illumina). Single-read sequencing was carried out on an Illumina HiScanSQ sequencer, using a HiSeq SBS v3 Kit to generate 50-base reads, with a target of approximately 10 million reads per sample. FASTQs were aligned to a human reference genome to generate gene counts.

### RNAseq data analysis

RNAseq data was analyzed in R version 3.0.1 using package edgeR [41–46]. Genes that were not expressed in at least 10% of the samples were excluded from analysis. To compare the data across different conditions, the raw counts were normalized as the number of counts per million (cpm) mapped for each sample. Next, 1 was added to all normalized data to allow taking ratio, stimulated over unstimulated samples (FC). Responders to the stimulations were identified as those with log2(FC) expression for 2 out of 3 genes selected as response indicator genes: CXCL9, CXCL10, and IFNG, ≥ log2(2). After estimating dispersions, a linear model was fit to determine genes responding differently to WNV in symptomatic vs asymptomatic groups. The model was y = μ + β1XS + β2XA:d10 + β3XA:d2 + β4XS:d2 + β5XA:d3 + β6XS:d3 + β7XA:d4 + β8XS:d4 +β9XA:d5 + β10XS:d5 + β11XA:d6 + β12XS:d6 + β13XA:d7 + β14XS:d7 + β15XA:d8 + β16XA:d9 + β17XA:WNV + β18XS:WNV +β19XA:Flu + β20XS:Flu + ε where μ is an estimates for log-expression level for asymptomatic unstimulated sample from asymptomatic donor 1, and d, S, A, WNV, and Flu are abbreviations for donor, symptomatic, asymptomatic, and peptides derived from WNV and flu, respectively. The genes are considered differentially expressed if absolute estimate of the log2(FC) corresponding to the effect ≥ log2(1.5) at false discovery rate (FDR) adjusted p-value ≤ 0.05. Principal component analysis was performed for the responders with WNV stimulation using the differentially expressed genes in log2(FC) values.

### Statistical analysis

Statistical comparisons between symptomatic and asymptomatic subjects were performed with the GraphPad Prism software version 5.0a (GraphPad Software, La Jolla, CA) using Student’s t test with Welch’s correction for two column comparisons and ANOVA analysis followed by Holm-Sidak's multiple comparisons test for multi-column comparisons.

### Ethics statement

The authors' respective Institutional Review Boards (the Benaroya Research Institute at Virginia Mason Institutional Review Board and UCSF Committee on Human Research) approved this study. All adult subjects provided informed written consent. The study did not include any participants who were children.

## Supporting Information

S1 Fig Identification of CD4+ T cell epitopes within WNV proteins. (DOCX)

S2 Fig Number and phenotype of WNV-specific Tin vitro stimulated cells (DOCX)

S3 Fig Functional profile of WNV-specific T cell lines. (DOCX)

S4 Fig Correlation between *ex vivo* phenotype and functional profile of WNV-specific T cell lines. (DOCX)

S1 Table WNV-specific T cell epitopes. (DOCX)
