# Supplementary material for: Neuroinvasive West Nile Infection Elicits Elevated and Atypically Polarized T Cell Responses That Promote a Pathogenic Outcome
Source: PLoS Pathog. 2016 Jan 21;12(1):e1005375. doi: 10.1371/journal.ppat.1005375 (PMC4721872; doi:10.1371/journal.ppat.1005375)
Supplement: S1 Fig — (DOCX) [file ppat.1005375.s001.docx]

**

**

S1 Fig. Identification of CD4+ T cell epitopes within WNV proteins. A) Staining of in vitro stimulated cells from a representative WNV infected subject with a HLA-DRB1*01:01 haplotype using tetramers (Tmer) loaded with peptide pools spanning the WNV envelope protein. Peptide pools 2, 4, 5, 6, 10, and 13 contained DRB1*01:01-restricted epitopes. B) Identification of the antigenic peptide within envelope pools 2 and 4 by staining in vitro stimulated cells again using tetramers (Tmer) loaded with single peptides. The pool 2 epitope was identified within the Env 39-56 peptide. The pool 4 epitope was identified within the Env 127-144 peptide.
