# Supplementary material for: Neuroinvasive West Nile Infection Elicits Elevated and Atypically Polarized T Cell Responses That Promote a Pathogenic Outcome
Source: PLoS Pathog. 2016 Jan 21;12(1):e1005375. doi: 10.1371/journal.ppat.1005375 (PMC4721872; doi:10.1371/journal.ppat.1005375)
Supplement: S2 Fig — (DOCX) [file ppat.1005375.s002.docx]

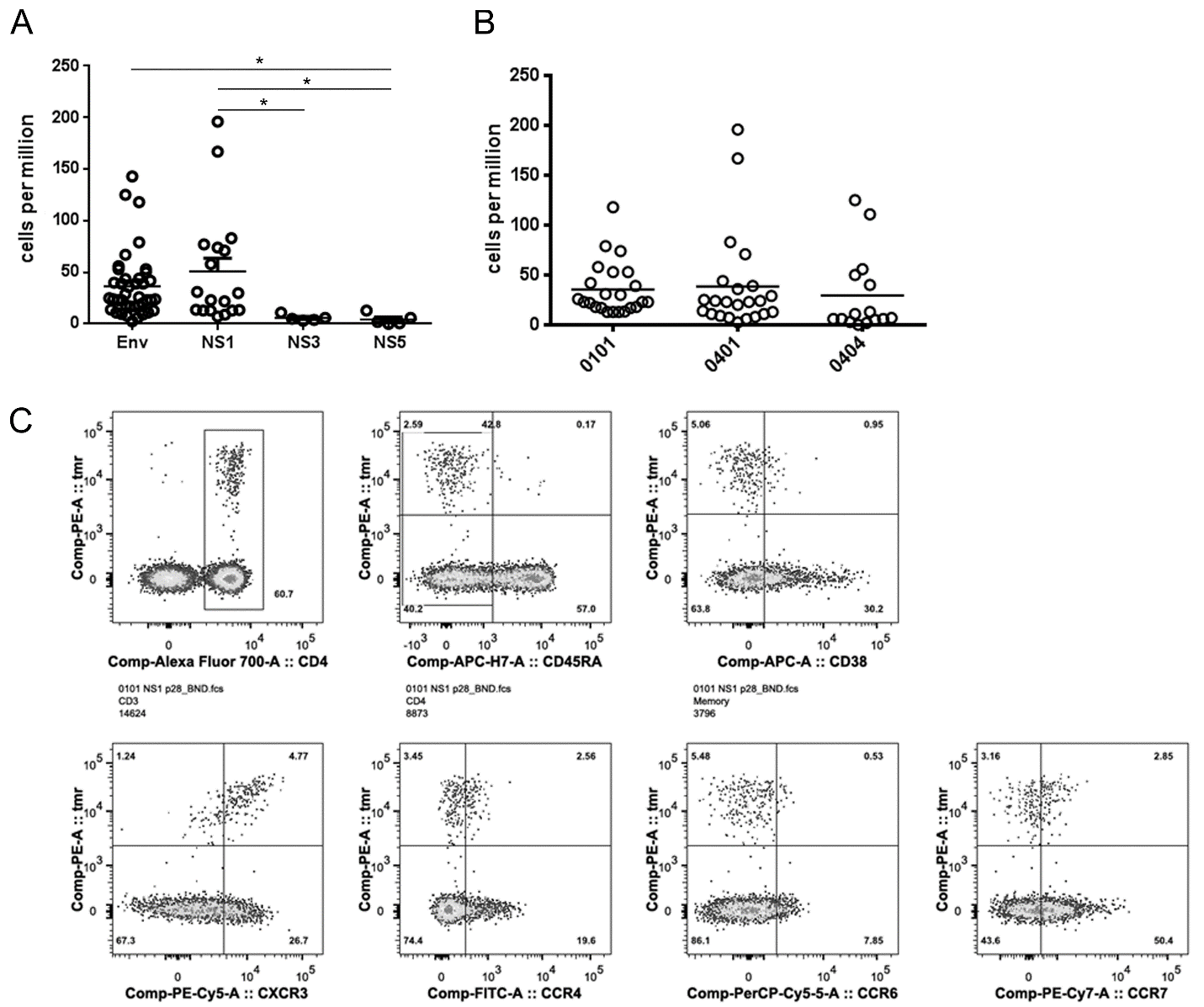


S2 Fig. Number and Phenotype of WNV-specific T cells. A) Direct *ex vivo* enumeration of WNV-specific T cells from WNV infected subjects with DRB1*01:01 (8 subjects), DRB1*04:01 (10 subjects), or DRB1*04:04 haplotypes (6 subjects). Each data point represents the frequency of T cells specific for a single WNV epitope (derived from the Env, NS1, NS3, or NS5 protein as indicated) measured in a single subject. B) Direct *ex vivo* enumeration of WNV-specific T cells from the WNV infected subjects indicated in panel A. Each data point represents the frequency of T cells specific for a single WNV epitope restricted by DRB1*01:01, DRB1*04:01, or DRB1*04:04. C) Representative Ex vivo tetramer staining and cell surface phenotyping for a representative WNV infected subject with a DRB1*01:01 haplotype using an NS1 205-220 tetramer (Tmr) and CD45RA, CD38, CXCR3, CCR4, CCR6, and CCR7 antibodies. Numbers in the upper right quadrant of each panel indicate the percentage of epitope specific cells that express that marker.
