# Supplementary material for: Neuroinvasive West Nile Infection Elicits Elevated and Atypically Polarized T Cell Responses That Promote a Pathogenic Outcome
Source: PLoS Pathog. 2016 Jan 21;12(1):e1005375. doi: 10.1371/journal.ppat.1005375 (PMC4721872; doi:10.1371/journal.ppat.1005375)
Supplement: S3 Fig — (DOCX) [file ppat.1005375.s003.docx]

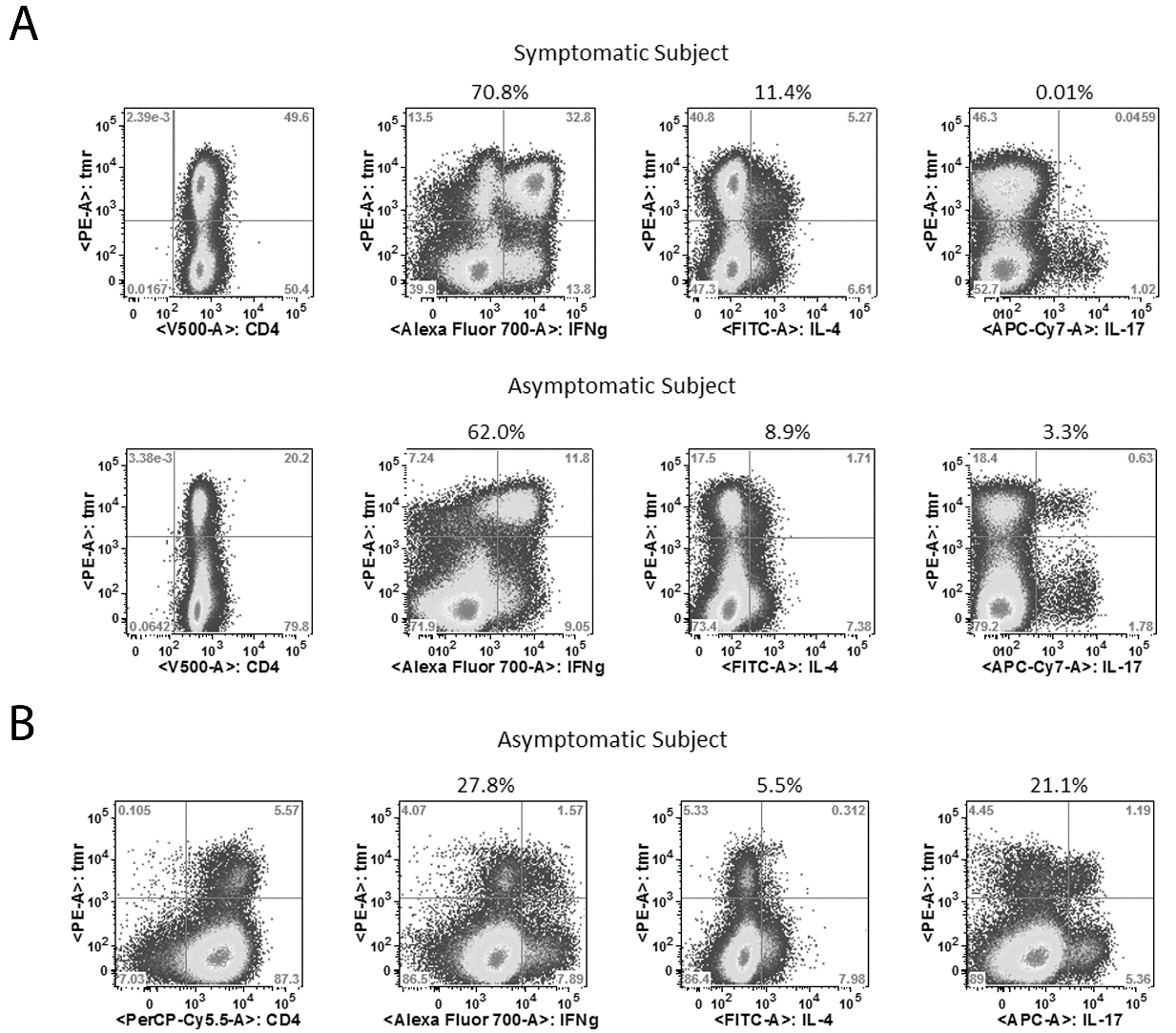


S3 Fig. Functional Profile of WNV-specific T cell lines. A) Intracellular cytokine staining results from representative WNV infected subjects with DRB1*01:01 haplotypes who had neuroinvasive (upper panels) or asymptomatic infections (lower panels) using an Env 127-144 tetramer (Tmr) and IFN-γ, IL-4, IL10, and IL-17 antibodies. Numbers in the upper right quadrant of each panel indicate the percentage of epitope specific cells that positive for the given cytokine. B) A limited number of ICS staining for WNV infected subjects utilized an alternative antibody panel. To avoid experimental bias, each set of experiments included an equal number of cell lines from who subjects had neuroinvasive or asymptomatic infections.
