# Supplementary material for: Neuroinvasive West Nile Infection Elicits Elevated and Atypically Polarized T Cell Responses That Promote a Pathogenic Outcome
Source: PLoS Pathog. 2016 Jan 21;12(1):e1005375. doi: 10.1371/journal.ppat.1005375 (PMC4721872; doi:10.1371/journal.ppat.1005375)
Supplement: S4 Fig — (DOCX) [file ppat.1005375.s004.docx]

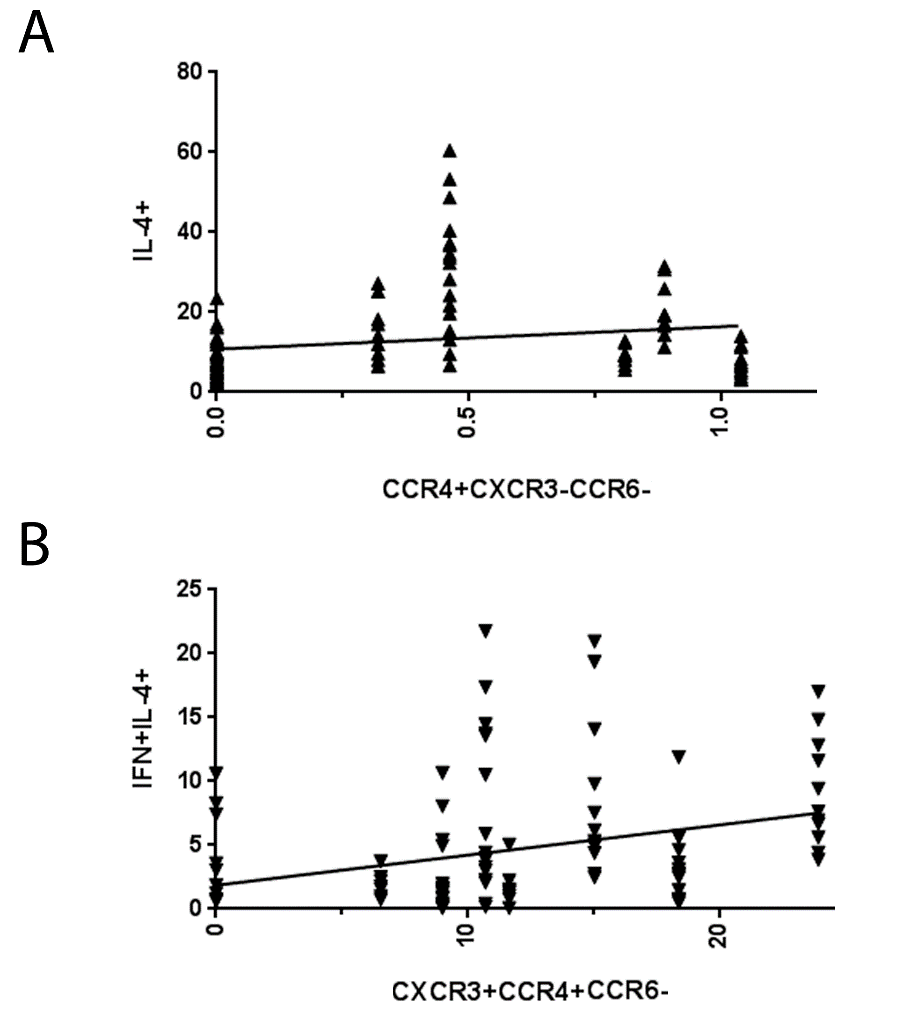


S4 Fig. Correlation between ex vivo phenotype and functional profile of WNV-specific T cell lines. A) Regression analysis indicated that the percentage of WNV specific cells with a Th2 surface phenotype (CCR4+CXCR3-CCR6-) was weakly correlated with the level of IL-4 production by WNV specific T cell lines from the same subject (p = 0.04). B) Regression analysis indicated that the percentage of WNV specific cells with a Th^ surface phenotype (CXCR3+CCR4+ CCR6-) was strongly correlated with co-production of IFN-γ and IL-4 by WNV specific T cell lines from the same subject (p = 0.002).
