# Supplementary material for: Neuroinvasive West Nile Infection Elicits Elevated and Atypically Polarized T Cell Responses That Promote a Pathogenic Outcome
Source: PLoS Pathog. 2016 Jan 21;12(1):e1005375. doi: 10.1371/journal.ppat.1005375 (PMC4721872; doi:10.1371/journal.ppat.1005375)
Supplement: S1 Table — (DOCX) [file ppat.1005375.s005.docx]

**Supplementary Table 1. WNV-Specific T cell Epitopes**^a^

| **Protein** | **Range** | **HLA-DR** | **Amino acid sequence** | **Positive Subjects** |
| --- | --- | --- | --- | --- |
| Cap | 77-95 | B1*01:01 | AMKHLLSFKKELGTLTSA | 3 of 4 |
| Cap | 16-33 | B1*01:03 | MLKRGMPRVLSLIGLKRA | 2 of 2 |
| Cap | 24-41 | B1*11:01 | VLSLIGLKRAMLSLIDGK | 2 of 2 |
| Cap | 77-95 | B1*11:01 | AMKHLLSFKKELGTLTSA | 2 of 2 |
| Cap | 24-41 | B1*11:04 | VLSLIGLKRAMLSLIDGK | 3 of 3 |
| Cap | 77-95 | B1*11:04 | AMKHLLSFKKELGTLTSA | 3 of 3 |
| Cap | 16-33 | B5*01:01 | MLKRGMPRVLSLIGLKRA | 4 of 5 |
| Cap | 77-95 | B5*01:01 | AMKHLLSFKKELGTLTSA | 4 of 5 |
| M | 37-53 | B1*01:01 | LRNPGYALVAAVIGWML | 2 of 4 |
| M | 44-61 | B1*03:01 | LVAAVIGWMLGSNTMQRV | 4 of 4 |
| M | 60-75 | B1*03:01 | RVVFVVLLLLVAPAYS | 3 of 4 |
| M | 22-38 | B1*07:01 | STKATRYLVKTESWILR | 2 of 4 |
| M | 29-46 | B1*07:01 | LVKTESWILRNPGYALVA | 2 of 4 |
| M | 29-46 | B1*15:01 | LVKTESWILRNPGYALVA | 2 of 4 |
| Env | 39-56 | B1*01:01 | PTIDVKMMNMEAANLAEV | 3 of 4 |
| **Env** | **127-144** | **B1*01:01** | **GRTILKENIKYEVAIFVH** | **4 of 4** |
| Env | 164-180 | B1*01:01 | AGRFSITPAAPSYTLKL | 2 of 4 |
| **Env** | **246-263** | **B1*01:01** | **HATKQSVIALGSQEGALH** | **4 of 4** |
| Env | 356-373 | B1*01:01 | VTVNPFVSVATANAKVLI | 2 of 4 |
| Env | 442-459 | B1*01:01 | VHQVFGGAFRSLFGGMSW | 2 of 4 |
| Env | 450-467 | B1*01:01 | FRSLFGGMSWITQGLLGA | 2 of 4 |
| Env | 203-219 | B1*01:03 | VMTVGTKTFLVHREWFM | 3 of 3 |
| Env | 32-48 | B1*03:01 | TIMSKDKPTIDVKMMNM | 2 of 4 |
| Env | 39-56 | B1*04:01 | PTIDVKMMNMEAANLAEV | 4 of 7 |
| Env | 54-71 | B1*04:01 | AEVRSYCYLATVSDLSTK | 5 of 7 |
| **Env** | **164-180** | **B1*04:01** | **AGRFSITPAAPSYTLKL** | **5 of 7** |
| Env | 246-263 | B1*04:01 | HATKQSVIALGSQEGALH | 5 of 7 |
| **Env** | **356-373** | **B1*04:01** | **VTVNPFVSVATANAKVLI** | **6 of 7** |
| Env | 390-407 | B1*04:01 | EQQINHHWHKSGSSIGKA | 4 of 7 |
| **Env** | **246-263** | **B1*04:04** | **HATKQSVIALGSQEGALH** | **5 of 5** |
| Env | 356-373 | B1*04:04 | VTVNPFVSVATANAKVLI | 5 of 5 |
| Env | 486-501 | B1*04:04 | LAVGGVLLFLSVNVHA | 3 of 5 |
| Env | 164-180 | B1*07:01 | AGRFSITPAAPSYTLKL | 3 of 4 |
| Env | 195-212 | B1*07:01 | GIDTNAYYVMTVGTKTFL | 3 of 4 |
| Env | 343-358 | B1*11:01 | VASLNDLTPVGRLVTV | 2 of 2 |
| Env | 377-393 | B1*11:01 | PPFGDSYIVVGRGEQQI | 2 of 2 |
| Env | 406-421 | B1*11:01 | KAFTTTLKGAQRLAAL | 2 of 2 |
| Env | 426-443 | B1*11:01 | WDFGSVGGVFTSVGKAVH | 2 of 2 |
| Env | 431-447 | B1*11:01 | VGGVFTSVGKAVHQVFG | 2 of 2 |
| Env | 434-451 | B1*11:01 | VFTSVGKAVHQVFGGAFR | 2 of 2 |
| Env | 343-358 | B1*11:04 | VASLNDLTPVGRLVTV | 3 of 3 |
| Env | 377-393 | B1*11:04 | PPFGDSYIVVGRGEQQI | 2 of 3 |
| Env | 406-421 | B1*11:04 | KAFTTTLKGAQRLAAL | 2 of 3 |
| Env | 356-373 | B1*15:01 | VTVNPFVSVATANAKVLI | 4 of 4 |
| Env | 465-482 | B1*15:01 | LGALLLWMGINARDRSIA | 3 of 4 |
| Env | 195-212 | B5*01:01 | GIDTNAYYVMTVGTKTFL | 5 of 5 |
| Env | 270-287 | B5*01:01 | IPVEFSSNTVKLTSGHLK | 4 of 5 |
| Env | 278-295 | B5*01:01 | TVKLTSGHLKCRVKMEKL | 5 of 5 |
| NS1 | 26-42 | B1*01:01 | EAWMDRYKYYPETPQGLA | 4 of 4 |
| **NS1** | **205-220** | **B1*01:01** | **RLNDTWKLERAVLGEVK** | **4 of 4** |
| NS1 | 26-42 | B1*01:03 | EAWMDRYKYYPETPQGLA | 2 of 2 |
| NS1 | 205-220 | B1*01:03 | RLNDTWKLERAVLGEVK | 2 of 2 |
| **NS1** | **205-220** | **B1*04:01** | **RLNDTWKLERAVLGEVK** | **6 of 7** |
| NS1 | 212-226 | B1*04:01 | LERAVLGEVKSCTWPETH | 6 of 7 |
| NS1 | 49-64 | B1*11:01 | AHKEGVCGLRSVSRLEH | 2 of 2 |
| NS1 | 56-72 | B1*11:01 | GLRSVSRLEHQMWEAVK | 2 of 2 |
| NS1 | 71-86 | B1*11:01 | VKDELNTLLKENGVDLSV | 2 of 2 |
| NS1 | 109-121 | B1*11:01 | EKLEIGWKAWGKSILFA | 2 of 2 |
| NS1 | 327-345 | B1*11:01 | SGCWYGMEIRPQRHDEK | 2 of 2 |
| NS1 | 49-64 | B1*11:04 | AHKEGVCGLRSVSRLEH | 2 of 3 |
| NS1 | 56-72 | B1*11:04 | GLRSVSRLEHQMWEAVK | 2 of 3 |
| NS1 | 71-86 | B1*11:04 | VKDELNTLLKENGVDLSV | 3 of 3 |
| NS1 | 205-220 | B5*01:01 | RLNDTWKLERAVLGEVK | 4 of 5 |
| NS2a | 25-42 | B1*01:03 | EVLRKRWTAKISMPAILI | 2 of 2 |
| NS2a | 123-140 | B1*15:01 | DARQILLWEIPDVLNSLA | 3 of 4 |
| NS2a | 83-100 | B5*01:01 | MATFKIQPVFMVASFLKA | 3 of 5 |
| NS2b | 107-124 | B1*01:01 | ISAYTPWAILPSVVGFWI | 3 of 4 |
| NS2b | 107-124 | B1*04:04 | ISAYTPWAILPSVVGFWI | 2 of 5 |
| NS3 | 29-46 | B1*01:01 | GLLGSYQAGAGVMVEGVF | 2 of 4 |
| NS3 | 91-107 | B1*01:01 | GQDEVQMIVVEPGKNVK | 2 of 4 |
| NS3 | 206-223 | B1*01:01 | PQIIKEAINRRLRTAVLA | 2 of 4 |
| NS3 | 273-289 | B1*01:01 | PHRVPNYNLFVMDEAHF | 2 of 4 |
| NS3 | 14-31 | B1*01:03 | KKGDTTTGVYRIMTRGLL | 2 of 2 |
| NS3 | 22-38 | B1*01:03 | VYRIMTRGLLGSYQAGA | 2 of 2 |
| NS3 | 206-223 | B1*01:03 | PQIIKEAINRRLRTAVLA | 2 of 2 |
| NS3 | 280-297 | B1*01:03 | NLFVMDEAHFTDPASIAA | 2 of 2 |
| NS3 | 596-612 | B1*01:03 | WIDARVYSDHQALKAFK | 2 of 2 |
| NS3 | 199-216 | B1*03:01 | GKTRRILPQIIKEAINRR | 3 of 4 |
| NS3 | 206-223 | B1*03:01 | PQIIKEAINRRLRTAVLA | 3 of 4 |
| NS3 | 280-297 | B1*03:01 | NLFVMDEAHFTDPASIAA | 2 of 4 |
| NS3 | 565-582 | B1*03:01 | FDGPRTNTILEDNNEVEV | 2 of 4 |
| NS3 | 596-612 | B1*03:01 | WIDARVYSDHQALKAFK | 3 of 4 |
| NS3 | 280-297 | B1*04:01 | NLFVMDEAHFTDPASIAA | 4 of 7 |
| **NS3** | **91-107** | **B1*04:04** | **GQDEVQMIVVEPGKNVK** | **3 of 5** |
| NS3 | 234-250 | B1*04:04 | EALRGLPIRYQTSAVPR | 3 of 5 |
| NS3 | 304-321 | B1*04:04 | KVELGEAAAIFMTATPPG | 4 of 5 |
| NS3 | 312-326 | B1*04:04 | AIFMTATPPGTSDPF | 4 of 5 |
| NS3 | 530-547 | B1*04:04 | ERKNFLELLRTADLPVWL | 4 of 5 |
| NS3 | 22-38 | B1*11:01 | VYRIMTRGLLGSYQAGA | 2 of 2 |
| NS3 | 78-95 | B1*11:01 | CYGGPWKLQHKWNGQDEV | 2 of 2 |
| NS3 | 146-162 | B1*15:01 | VIGLYGNGVIMPNGSYI | 4 of 4 |
| NS3 | 206-223 | B1*15:01 | PQIIKEAINRRLRTAVLA | 4 of 4 |
| NS3 | 343-360 | B1*15:01 | RAWNSGYEWITEYTGKTV | 2 of 4 |
| NS3 | 351-368 | B1*15:01 | WITEYTGKTVWFVPSVKM | 2 of 4 |
| NS3 | 405-422 | B1*15:01 | FVITTDISEMGANFKASR | 2 of 5 |
| NS4a | 62-79 | B1*01:01 | VMTMGVFFLLMQRKGIGK | 2 of 4 |
| NS4a | 94-110 | B5*01:01 | CWMAEVPGTKIAGMLLL | 2 of 5 |
| NS4b | 179-196 | B1*01:01 | ILVSLAAVVVNPSVKTVR | 4 of 4 |
| NS4b | 56-72 | B1*03:01 | LKHLITSDYINTSLTSI | 2 of 4 |
| NS4b | 30-47 | B1*04:04 | GEFLLDLRPATAWSLYAV | 2 of 5 |
| NS4b | 179-196 | B1*04:04 | ILVSLAAVVVNPSVKTVR | 3 of 5 |
| NS4b | 209-226 | B1*07:01 | LWENGASSVWNATTAIGL | 2 of 4 |
| NS4b | 133-150 | B1*11:04 | AMRSAQRRTAAGIMKNAV | 2 of 3 |
| NS4b | 241-255 | B1*11:04 | TWTLIKNMEKPGLKR | 2 of 3 |
| NS4b | 117-134 | B5*01:01 | LLFCHYAYMVPGWQAEAM | 4 of 5 |
| NS4b | 233-250 | B5*01:01 | GWLSCLSITWTLIKNMEK | 4 of 5 |
| NS4b | 241-255 | B5*01:01 | TWTLIKNMEKPGLKR | 4 of 5 |
| NS5 | 462-479 | B1*01:01 | KKPGEFGKAKGSRAIWFM | 3 of 4 |
| NS5 | 470-486 | B1*01:01 | AKGSRAIWFMWLGARFL | 3 of 4 |
| NS5 | 613-631 | B1*01:01 | NTFTNLAVQLVRMMEGEGV | 3 of 4 |
| NS5 | 622-639 | B1*01:01 | LVRMMEGEGVIGPDDVEK | 3 of 4 |
| NS5 | 771-788 | B1*01:01 | YFHRRDLRLMANAICSAV | 2 of 4 |
| NS5 | 7-24 | B1*01:03 | TLGEVWKERLNQMTKEEF | 2 of 2 |
| NS5 | 220-235 | B1*01:03 | YWVSRASGNVVHSVNM | 2 of 2 |
| NS5 | 302-319 | B1*01:03 | YRTWNYHGSYDVKPTGSA | 2 of 2 |
| NS5 | 394-411 | B1*01:03 | RMCSREEFIRKVNSNAAL | 2 of 2 |
| NS5 | 712-729 | B1*01:03 | PFCSNHFTELIMKDGRTL | 2 of 2 |
| NS5 | 31-45 | B1*03:01 | AIIEVDRSAAKHARK | 2 of 4 |
| NS5 | 630-647 | B1*03:01 | GVIGPDDVEKLTKGKGPK | 3 of 4 |
| NS5 | 249-266 | B1*04:01 | WKGPQYEEDVNLGSGTRA | 2 of 7 |
| NS5 | 529-546 | B1*04:01 | GGKIYADDTAGWDTRITR | 2 of 7 |
| NS5 | 704-721 | B1*04:01 | GWYDWQQVPFCSNHFTEL | 2 of 7 |
| NS5 | 771-788 | B1*04:01 | YFHRRDLRLMANAICSAV | 3 of 7 |
| NS5 | 75-92 | B1*04:04 | GKVIDLGCGRGGWCYYMA | 2 of 5 |
| **NS5** | **159-174** | **B1*04:04** | **HRTIRVLEMVEDWLHR** | **4 of 5** |
| NS5 | 226-243 | B1*04:04 | SGNVVHSVNMTSQVLLGR | 2 of 5 |
| NS5 | 336-351 | B1*04:04 | TITNVTTMAMTDTTPF | 2 of 5 |
| NS5 | 394-411 | B1*04:04 | RMCSREEFIRKVNSNAAL | 3 of 5 |
| NS5 | 402-421 | B1*04:04 | IRKVNSNAALGAMFEEQNQW | 3 of 5 |
| NS5 | 771-788 | B1*04:04 | YFHRRDLRLMANAICSAV | 3 of 5 |
| NS5 | 870-887 | B1*04:04 | VAINQVRAIIGDEKYVDY | 3 of 5 |
| NS5 | 212-229 | B1*11:01 | SRNSTHEMYWVSRASGNV | 2 of 2 |
| NS5 | 279-296 | B1*11:01 | IKNRIERLRREYSSTWHH | 2 of 2 |
| NS5 | 318-334 | B1*11:01 | SASSLVNGVVRLLSKPW | 2 of 2 |
| NS5 | 448-463 | B1*11:01 | ECHTCIYNMMGKREKK | 2 of 2 |
| NS5 | 514-531 | B1*11:01 | LQKLGYILREVGTRPGGK | 2 of 2 |
| NS5 | 23-40 | B1*11:04 | EFTRYRKEAIIEVDRSAA | 2 of 3 |
| NS5 | 31-45 | B1*11:04 | AIIEVDRSAAKHARK | 2 of 3 |
| NS5 | 212-229 | B1*11:04 | SRNSTHEMYWVSRASGNV | 3 of 3 |
| NS5 | 271-288 | B1*11:04 | LLNSDTSKIKNRIERLRR | 2 of 3 |
| NS5 | 279-296 | B1*11:04 | IKNRIERLRREYSSTWHH | 3 of 3 |
| NS5 | 318-334 | B1*11:04 | SASSLVNGVVRLLSKPW | 3 of 3 |
| NS5 | 514-531 | B1*11:04 | LQKLGYILREVGTRPGGK | 3 of 3 |
| NS5 | 574-590 | B1*11:04 | TYRHKVVKVMRPAADGR | 2 of 3 |
| NS5 | 115-132 | B1*15:01 | LVQSYGWNIVTMKSGVDV | 2 of 4 |
| NS5 | 771-788 | B1*15:01 | YFHRRDLRLMANAICSAV | 3 of 4 |
| NS5 | 380-395 | B5*01:01 | TNWLWAFLAREKRPRM | 3 of 5 |
| NS5 | 448-463 | B5*01:01 | ECHTCIYNMMGKREKK | 3 of 5 |
| NS5 | 644-660 | B5*01:01 | KGPKVRTWLFENGEERL | 2 of 5 |
| NS5 | 878-894 | B5*01:01 | IIGDEKYVDYMSSLKRY | 3 of 5 |

^a^ Epitopes that were chosen for extensive ex vivo tetramer analysis are listed in boldface
